# Salidroside inhibits melanin synthesis and melanoma growth via mTOR and PI3K/Akt pathways

**DOI:** 10.3389/fonc.2025.1583580

**Published:** 2025-07-10

**Authors:** Qi Ouyang, Shengye Tian, Hengyu Zhou, Ying Mao, Xiang Li, Feng Yan, Ailong Liu, Xiang Hu, Changqiao You, Jun He

**Affiliations:** ^1^ Hunan Provincial Key Laboratory of Regional Hereditary Birth Defects Prevention and Control, Changsha Hospital for Maternal & Child Health Care Affiliated to Hunan Normal University, Hunan Normal University, Changsha, China; ^2^ The National and Local Joint Engineering Laboratory of Animal Peptide Drug Development, College of Life Sciences, Hunan Normal University, Changsha, China; ^3^ Hunan Provincial Engineering Technology Research Center of Stem Cell Exosome, Hunan Landfar Biotechnology Co. Ltd., Changsha, China

**Keywords:** network pharmacology, salidroside, melanoma, oxidative stress, PI3K/Akt/mTOR pathway

## Abstract

**Background:**

Melanomas are caused by the malignant transformation of melanocytes. Numerous studies have demonstrated that the tyrosol components of salidroside inhibit tyrosinase activity. The PI3K/Akt/mTOR signaling pathway plays a crucial role in biological pigment synthesis. However, how salidroside achieves its anti-melanoma effect in melanoma by regulating PI3K/Akt/mTOR remains poorly understood. This study aimed to explore the effect of salidroside on PI3K/Akt/mTOR in melanoma, which plays a role in regulating melanogenesis.

**Methods:**

Network pharmacology was predicted that salidroside may exert an anti-melanoma effect through modulating melanin synthesis functions and signaling pathways. Zebrafish whole-embryo *in situ* hybridization, RT-qPCR, melanin synthesis and tumorigenesis assays, and were performed to investigate the therapeutic efficacy of salidroside in melanin synthesis. The mechanism of salidroside in anti-melanoma activity was examined by RT-qPCR, Western blot, immunofluorescence, *in vivo* imaging, immunohistochemistry.

**Results:**

We confirmed salidroside may exert an anti-melanoma effect through modulating melanin synthesis-related gene expression and PI3K/Akt pathway by Network pharmacology. Furthermore, salidroside slowed melanin synthesis in zebrafish embryos and H_2_O_2_-induced B16F10 cells by inhibited oxidative stress. Moreover, we determined the effect of salidroside on anti-melanin synthesis via PI3K/Akt/mTOR pathway *in vitro*, and western blot results showed that salidroside increased the expression of Nrf2 in the nucleus, as well as inhibited the phosphorylation of mTOR and PI3K/Akt pathway. Finally, intratumoral administration showed salidroside suppressed melanoma growth.

**Conclusion:**

Salidroside inhibits melanin synthesis and melanoma development most likely by its antioxidant properties and downregulating the PI3K/Akt/mTOR pathway. Our results may provide a novel therapeutic strategy for the treatment of melanoma.

## 1 Introduction

Melanoma, a malignant tumor arising from melanocytes, is one of the most aggressive and lethal forms of skin cancer, and is characterized by its invasive and metastatic potential (1).

Unlike benign melanocytic naevi, melanomas exhibit uncontrolled proliferation and survival signals that often lead to fatal outcomes (2). Melanin has been shown to act as a natural photoprotective agent, shielding cells from ultraviolet (UV) radiation-induced DNA damage and oxidative stress (3, 4). Moreover, research has shown that the presence of melanin in metastatic melanoma cells decreases the efficacy of radiotherapy (5). Recent findings suggest that lysophosphatidic acid (LPA) is produced by melanoma, influencing melanin synthesis and modulating tumor immunity through cytokine and chemokine release, thereby affecting cancer progression and metastasis (6). For instance, melanin can alter the local pH, creating an acidic environment that favors tumor metastasis and invasion (7, 8). Recent research has demonstrated that the inhibition of melanogenesis in melanoma cells can hamper their metastatic potential (9, 10). This is partly because melanin-synthesis pathways are intricately linked to other cellular processes, such as epithelial–mesenchymal transition (EMT), which is a key driver of metastasis (11). Researchers have observed the suppression of EMT-related markers by downregulating melanogenic enzymes, such as tyrosinase, suggesting a potential therapeutic avenue for targeting melanogenesis to curb metastasis (12). In conclusion, research from the 1990s to 2024 has consistently highlighted melanin’s role in melanoma immune escape and therapeutic resistance. However, ongoing studies continue to uncover how manipulating melanin could potentially be leveraged to improve melanoma treatment outcomes. Thus, melanogenesis is emerging as a critical therapeutic target in melanoma treatment.

Salidroside is extracted from the perennial herb *Rhodiola rosea*, and its main medicinal constituents are salidroside and tyrosol (13). Previous research has shown that this herb not only possesses anti-tumor properties but also exerts antioxidant and anti-aging effects (14). Aging is a gradual process characterized by skin sagging, the appearance of pigmentation spots, and other symptoms. Consequently, many cosmetic products incorporate herbal ingredients with antioxidant properties to combat signs of aging and dullness (15, 16). Pigmentation processes in melanocytes are often influenced by oxidative stress caused by UV exposure or other environmental factors. Salidroside has been shown to combat oxidative damage and slow down aging-associated processes. Exploring its role in inhibiting melanogenesis aligns with its anti-aging potential, as it could help reduce pigmentation spots and improve skin clarity (17).

Melanin production is primarily regulated by the activity of the enzyme tyrosinase, a key player in the melanogenesis pathway (18). Tyrosinase, a key enzyme in melanin synthesis, exhibits a highly conserved structure across species and exists in various high-molecular-weight forms that play crucial roles in regulating melanogenesis. These forms include unmodified and glycosylated variants, which are essential for enzyme function and transport to melanosomes (19). Regulation of tyrosinase activity is influenced by various intracellular signaling pathways, including the cAMP/PKA and MAPK pathways (20). These pathways are crucial for stimulating melanin synthesis in melanocytes following exposure to sunlight or other stimuli.

The PI3K/AKT/mTOR signaling pathway plays a vital role in cell growth, metabolism, and survival (21). Hyperactivation of the PI3K/AKT/mTOR axis may promote cell proliferation, enhance cell survival, and confer resistance to apoptosis (22). Understanding the interplay between melanogenesis and the PI3K/AKT/mTOR pathway may provide insights into therapeutic targets for melanoma treatment. In our ongoing study, we found that salidroside inhibits tyrosinase activity and cellular melanin production. The findings of the present study showed that salidroside inhibits melanin formation via the PI3K/AKT/mTOR signaling pathway.

The objective of the present study was to analyze the composition of salidroside and identify common targets of salidroside in melanoma using network pharmacology analysis combined with experimental verification. We investigated the anti-melanoma effects of salidroside in B16F10 cells, zebrafish, and mouse models, and examined the involvement of PI3K/Akt/mTOR signaling in these effects. Our findings indicated that salidroside directly downregulated the gene expression involved in melanin synthesis and suppressed the growth of melanoma by inhibiting PI3K/Akt/mTOR pathway. These findings provide a theoretical basis for the development of salidroside as a novel candidate in melanoma-targeted therapy.

## 2 Methods

### 2.1 Drug and antibodies

Salidroside was obtained from Aladdin (Cat:10338-51-9, Shanghai, China) with a purity of ≥ 98%, as determined by high-performance liquid chromatography (HPLC). Antibodies: TYR (Immunoway, YM6968), MITF (Immunoway, YT2769), TRP1 (Immunoway, YN8781). β-actin (Abclonal, AC062), GAPDH (Abclonal, A19056) and β-Tubulin (Abclonal, AC015). mTOR and p-mTOR antibodies (Zenbio, R380411, R25033). Akt and p-Akt (Immunoway, YT0185, YP006). PI3K (Santa, sc-376112). miTF (Servicebio, GB111419-100). Ki-67 (Origene, TA801577). Nrf2 (Cell Signaling Technology, 12721S) Rabbit IgG-HRP (Abclonal, AS030). Mouse IgG-HRP (Abclonal, AS061). The DIG mixture (Thermo Fisher, Q152P.9900). RPMI Media 1640 (Gibco, Cat: 11875093). Fetal bovine serum (FBS) and 1% penicillin and streptomycin (Gibco, USA). MTT (Sigma, USA). Trypsin was produced by our laboratory. Rapamycin was purchased from Selleck (Cat: S1039).

### 2.2 Construction of oxidative-stress model

To establish an oxidative stress model in B16F10 melanoma cells, we used H_2_O_2_ (Sangong, Shanghai, China) for a specific treatment duration. Multiple studies have demonstrated that H_2_O_2_ concentrations ranging from 25 μM to 1mM is within a commonly accepted range for inducing oxidative stress without causing excessive cytotoxicity in B16F10 cells (23). Different concentrations of H_2_O_2_ were prepared, and cells were incubated for 3–4 h and the culture medium was exchanged with RPMI 1640 medium (Thermo Fisher, 11875093). The optimal concentration was determined using a cell-viability assay to construct an oxidative-stress model.

### 2.3 Cell culture and cell-viability assay

B16F10 cells were purchased from American Type Culture Collection) (ATCC; Shanghai, China). Cells were cultured in RPMI 1640 medium containing 10% fetal bovine serum (FBS) and 1% penicillin–streptomycin. Cells were cultured in a constant temperature incubator under 5% CO_2_ at 37˚C. To examine cell viability, we used 3-(4, 5-Dimethylthiazol-2-yl)-2, 5-diphenyltetrazolium bromide (MTT) (Sangong, Shanghai). After addition of 5 mg/mL MTT, the cells were incubated for 4 h; 100 μL of dimethyl sulphoxide (DMSO) (Sangong, Shanghai) lysed the formazan formed by the cells, and optical density (OD) was measured at 492 nm using an enzyme Labelling Instrument (PL-9602, Perlong Medical, Beijing, China). Cell viability was expressed as a percentage of the control cells.

### 2.4 Assay for melanin content

Melanin content was determined as described by Ozeki et al. (24). Sodium hydroxide (1M NaOH) is a strong alkali that disrupts cellular components and dissolves melanin by breaking down protein complexes and cell membranes. Heating the sample at 65°C enhances the solubilization process by increasing the efficiency of pigment release. Once melanin is dissolved in NaOH, it exhibits a characteristic absorbance at 492 nm. By measuring the absorbance using a microplate reader at this wavelength, the amount of melanin can be quantified (25). B16F10 cells were treated with salidroside or H_2_O_2_, washed with phosphate-buffered saline (PBS), collected, and subjected to melanin content assay. The collected cell pellets were dissolved in 1M NaOH (Sangong, Shanghai) (65°C) for 1 h, and colorimetric photometric analysis was performed at 492 nm with a microplate reader. Melanin content was expressed as the ratio of the salidroside-treated group to the control group (% of the control group).

### 2.5 Cellular tyrosinase activity assay

The Tyrosinase (Tyr) Activity Assay Kit, Colorimetric Method (Sangon Biotech, Shanghai) is used to measure the activity of tyrosinase, an enzyme involved in melanin biosynthesis. Tyrosinase catalyzes the hydroxylation of L-tyrosine to form L-DOPA, and the oxidation of L-DOPA to dopachrome, a precursor in melanin synthesis. In this assay, a substrate like L-DOPA or L-tyrosine reacts with tyrosinase to produce dopachrome, resulting in a color change that can be measured spectrophotometrically. The color intensity, usually at 490–510 nm, is directly proportional to the enzyme activity, allowing for quantitative analysis. This assay is commonly used in skin pigmentation research, cosmetic product testing, and pharmaceutical screening for tyrosinase inhibitors or activators (26).

B16F10 cells were cultured in 6-well plates, and treated in the same manner as described above. The cell suspension was centrifuged at 12,000 *g* for 10 min at 4°C to remove cellular debris, and then lysed using an ultrasonic Cell Grinder SCIENTZ-ID (SCIENTZ, China). Sonication was performed on ice using a pulse cycle of 3s on/10 s off and was repeated 30 times. The lysates were clarified by centrifugation at 12,000 g for 10 min at 4°C. The supernatants were carefully collected and immediately placed on ice. A tyrosinase (Tyr) activity assay kit and colorimetric method was used to examine the activity of the *in vitro* tyrosinase. The reaction mixture was prepared according to the kit and 100 μL was added to each well of a 96-well plate, repeated to 5 wells. After incubation at 37°C, the absorbance was measured at 505 nm. The percentage of tyrosinase activity was calculated as 52.5/10^6^ (sample OD/control OD).

### 2.6 Cell wound healing

B16F10 cells were seeded in 6-well plates (Servicebio, Wuhan, China). When 100% fusion was attained, the cells were scratched onto the plate using a 10 μL pipette tip. After washing twice with PBS, the cells were cultured in serum-free 1640 medium. For each sample, three areas were randomly selected and observed at 10 × 10 magnification at 0 h and 24 h. The relative distance was used to evaluate cell-migration ability using ImageJ.

### 2.7 Transwell migration assay

Cells were seeded at a density of 1 × 10^5^ onto the upper chamber with 200 μL serum-free medium, and 300 μL of medium supplemented with 10% FBS was added to the lower chamber. The cells were then allowed to migrate for 24 h. The cells were fixed with 4% paraformaldehyde for 15 min. The chamber was then washed with excess double-distilled water, dried at 24°C - 26°C (RT) for 24 h, and photographed under a microscope. The cells were counted using ImageJ software.

### 2.8 Western blot analysis

Western blotting was performed as described previously (27). Proteins were extracted from B16F10 cells using radioimmunoprecipitation assay (RIPA) lysis buffer (cat. G2002, Wuhan Servicebio, China) and a Protein Phosphatase Inhibitor (cat. P1260, Beijing Solarbio). Proteins were separated by 6% or 10% sodium dodecyl sulfate-polyacrylamide gel electrophoresis and transferred to polyvinylidene fluoride (PVDF) membranes activated with methanol. Membranes were sealed with 5% protein-rich skim milk at RT for 2h, followed by overnight incubation at 4°C with antibodies. After washing, the membranes were incubated with rabbit or mouse IgG-horseradish peroxidase (HRP) for 1h at RT. The proteins were visualized with electrochemiluminescence (ECL) (cat. P10060, Suzhou NCM). Protein bands were scanned using a Tanon 1600 Series Multifunctional Gel Image Analysis System, and band density was analyzed using ImageJ.

### 2.9 Mice

For *in vivo* studies, a syngeneic tumor transplantation model was established in BALB/c mice. Male mice (BALB/c) with an average weight of approximately 20 g, were purchased from Tianqin (Hunan, China). The animals were kept in isolation cages under 12 h light/dark cycles. Hindleg tumor homografts were established using a mouse B16F10 cell line. B16F10 cells (1.5 × 10^6^) in 100 μL PBS were implanted subcutaneously in the right hind leg of each mouse. After the tumors were allowed to grow to about 300 mm^3^, 4 or 8 mg/kg salidroside were infused into the mice tumors in the treatment group for 5 days. The control mice were intratumorally infuse d with the same dose of PBS. The mice were examined daily for changes in body weight and tumor area until the tumor volume overed 1,200 mm^3^. Tumor volume was calculated using the following formula: volume = ((width)^2^ × length) /2. Throughout the experiment, no signs of toxicity such as weight loss or behavioral abnormalities were observed, indicating that salidroside at 4 and 8 mg/kg is safe for intratumoral administration (28). All animal experiments were conducted in accordance with the guidelines of the Institutional Ethics Committee of the Medical College of Hunan Normal University (2024-776).

### 2.10 Immunohistochemistry and Immunofluorescence staining

The melanoma tissues were fixed and frozen rapidly. Tissue samples were then section into 6 μm slides to incubate with a primary antibody (Servicebio, Wuhan) MiTF overnight at 4°C. Imaging was performed under a microscope after incubation with a secondary antibody (Servicebio, Wuhan). The cells were stained using an AEC (3-amino-9-ethylcarbazole) Peroxidase Substrate Kit Chromogenic Kit (Solarbio, China, A2010), which provides a red staining product. Finally, the nuclei were stained with hematoxylin. The mounted sections were observed and images were captured using an inverted fluorescence microscope.

B16F10 cells were seeded into 12-well plates (Servicebio, Wuhan, China). The same previously described treatment and polarization protocols were used for these experiments. The cells were fixed using 4% paraformaldehyde (Servicebio, Wuhan, G1101), infiltrated with 0.2% Triton X-100 (Sigma, CAS#9036-19-5), and blocked with 5% BSA for 30 min. The cells were then incubated with primary antibodies specific for Nrf2 (1:200, Cell Signaling Technology, 12721S) and the slides incubated with Ki-67 (Immunoway, YM 8189) and mTOR (1:200, Zenbio, R380411) Thereafter, the cells were incubated with fluorescence-conjugated secondary antibodies (1:1000, Invitrogen) and mounted with a fluorescent mounting medium containing 4′,6-diamidino-2-phenylindole (DAPI) (Biosharp, BL739A). Images were captured using a confocal microscope (EVOSTM M7000; Thermo Fisher Scientific).

### 2.11 Hematoxylin and eosin staining

Slides were stained with hematoxylin and eosin (H&E) at RT (hematoxylin staining for 3 min and eosin staining for 15 s) for histopathology. Pathological changes in the colonic mucosa were observed under a light microscope.

### 2.12 Zebrafish strains

TU strain zebrafish (Danio rerio) were obtained from the China Zebrafish Resource Center (Wuhan, China). All wild-type zebrafish were maintained in housing systems (ESEN, Beijing, China) under 14 h/10 h day/night cycle at 27°C to 28°C. Adult male and female zebrafish were mated at a 1:1/1:2 ratio to obtain embryos and staged at hours post-fertilization (hpf). All zebrafish embryos were raised and maintained according to standard protocols, as previously described (29).

We assessed the survival of embryos daily by counting the number of live and dead embryos. Live embryos exhibit movement and normal morphology, while dead embryos appear opaque and immobile. At 24 hpf, we examined the embryos under a microscope for morphological abnormalities, including spinal curvature, edema, and other deformities.

### 2.13 *In situ* hybridization

Zebrafish *in situ* hybridization experiments were performed according to a previously reported standard procedure (30). The T7 promoter sequence was added to the 5’ end of the reverse primer used to synthesize the anti-sense RNA probes, which were synthesized using T7 RNA polymerase (EP0111, Thermo, Waltham, MA, USA) and DIG RNA Labelling Mix (11277073910, Roche, Basel, Switzerland). The images were captured using a Leica M205 FA fully automated fluorescence stereomicroscope (1802732S; Leica, Germany). The primer sequences used for the synthesis of anti-sense RNA probes are listed in **Table 1**.

**Table 1:** *In situ* hybridization probe for early zebrafish embryos.

Probe name	Amplification primers	Length
*oca2*	F: AGCAGCACATCTGCATCACT	641bp
	R: AGCTGGACACCTACCATCCT	
*try*	F: CATGCACAGATTCCGTGTGG	378bp
	R: GACGTCATTGTCCAGGGTGT	

### 2.14 RNA extraction and reverse transcription PCR

RT-qPCR was used to measure gene-transcription levels. Total RNA was extracted using RNAiso Plus (9108, Takara Shiga, Japan). cDNA was synthesized from 1 μg of total RNA using NovoScripts plus All-in-one 1st Strand cDNA Synthesis Super Mix (E401; Novoprotein, Suzhou, China), following the manufacturer’s instructions. Real-time RT-PCR analysis was performed on a CFX Connect PCR detection system (Bio-Rad, Germany) using the NovoStart Universal Fast SYBR qPCR Super Mix (E047, Novoprotein, Suzhou, China). In the present study, *β-actin* was used as the internal reference gene to detect relative gene expression. Relative gene-expression levels were normalized to *β-actin* mRNA expression and analyzed using the comparative Cq method (ΔΔCq). The same thermocycling conditions were applied for all primer sets: 95°C for 30 s, followed by 95°C for 30 s and 60°C for 30 s for 40 cycles. The primer sequences are listed in **Table 2**. Gene expression levels were quantified using quantitative PCR (qPCR). The gene expression was normalized to the negative control (NC), which was set to a relative expression value of 1.0. All values are presented as mean ± SD.

**Table 2:** RT-qPCR primers for genes associated with melanin synthesis in zebrafish or B16F10.

Gene name	Amplification primers	Length
*try*	F: GGGTGAGCACAACATTGACG	120bp
	R: CGTAGCTGTTGATTTGGGCG	
*oca2*	F: GCAACTGATAGCAAGCAGCC	104bp
	R: ATAGGTGGGCCTTCCGTAGT	
*dct*	F: GACTCTGTGTAACGGCACCA	105bp
	R: AGGTTCAGGCAGCTTCTCAC	
*tryp1a*	F: GGGAACTACAGCGGGTTTGA	98bp
	R: ATCGGCCACAGTCACTTACC	
*β-actin*	F: CGTGACATCAAGGAGAAG	147bp
	R: GAGTTGAAGGTGGTCTCAT	
β-Actin	F: ATGACCCAAGCCGAGAAGG	185bp
	R: CGGCCAAGTCTTAGAGTTGTTG	
Oca2	F: ATGCGCCTAGAGAACAAAGAC	225bp
	R: TAGCAGGTTTGACGGTCAGC	
Mitf	F: ACTTTCCCTTATCCCATCCACC	143bp
	R: TGAGATCCAGAGTTGTCGTACA	
Tyr	F: CTCTGGGCTTAGCAGTAGGC	107bp
	R: GCAAGCTGTGGTAGTCGTCT	
Nrf2	F:TCTTGGAGTAAGTCGAGAAGTGT	140bp
	R: GTTGAAACTGAGCGAAAAAGGC	
Keap1	F: TGCCCCTGTGGTCAAAGTG	104bp
	R: GGTTCGGTTACCGTCCTGC	

### 2.15 Determination of zebrafish and cell model SOD, O2•−, CAT, GSH and GSH-Px

According to the manufacturer’s instructions, the activity of superoxide dismutase (SOD), O_2_^•−^ content, catalase activity, GSH content and GSH-Px activity were measured using Total Superoxide Dismutase (T-SOD) assay kit (Hydroxylamine method) (Nanjing Jiancheng, A001-1-2), Inhibition and produce superoxide anion assay kit (Nanjing Jiancheng, A052-1-1), Catalase (CAT) assay kit (Visible light) (Nanjing Jiancheng, A007-1-1), Glutathione (GSH, Nanjing Jiancheng, A006-2-1) and Glutathione peroxidase (GSH-px, Nanjing Jiancheng, A005-1-2). Briefly, cells, and blood serum were collected in 1.5 mL EP tubes. Antioxidant assays were performed using individually homogenized embryos (n=8 per group) to ensure accurate measurement of enzymatic activities (SOD, CAT and O_2_^•−^). Cells were disrupted by ultrasound, the time being 10 s, the interval 6 s, and the number of ultrasonic times being 5 times. Thereafter, centrifugation was carried out at 2,000 g at 4°C for 5 min, and the supernatant was collected. Mice blood serum samples could be tested directly.

### 2.16 The prediction of therapeutic targets of salidroside against melanoma

SwissADME (http://www.swissadme.ch, accessed on 19 July 2022; Swiss Institute of Bioinformatics, Lausanne, Switzerland) is a web-based tool designed to generate physicochemical descriptors, including molecular formula, molecular weight, molar refractivity, atom type counts, polar surface area, lipophilicity, and water solubility (31). Additionally, it calculates pharmacokinetic properties, which encompass the study of drug absorption, distribution, metabolism, and excretion (ADME) within the body, all of which significantly influence a drug’s efficacy and safety. The pharmacokinetic properties evaluated by SwissADME include human gastrointestinal absorption, Caco-2 permeability, skin permeability coefficients, and interactions with cytochrome P450 (CYP) enzymes. The simplified molecular input line entry system (SMILES) format of salidroside, generated from Convert 3D model molecules into SMILES in Novo Pro (https://www.novopro.cn/tools/mol2smiles.html), was entered into the SwissADME.

The search terms “melanoma” or “melanogenesis” or “melanin” were used to search for therapeutic targets related to salidroside in GeneCards (https://www.genecards.org/), Online Mendelian Inheritance in Man (OMIM) (https://omim.org/), and DisGeNET (v7.0) (https://www.disgenet.org/). Overlapping targets between the disease and drug were screened and generated using a Venn diagram (https://www.bioinformatics.com.cn). Using the STRING website (https://cn.string-db.org/), we constructed a protein interaction network for overlapping genes. A minimum interaction score threshold of 0.700 was set to ensure high confidence. A cluster analysis was conducted based on the top 14 key targets.

### 2.17 Statistical analysis

Enrichment dot bubble was plotted by https://www.bioinformatics.com.cn (last accessed on 1st April 2025), an online platform for data analysis and visualization. All data are presented as the mean ± SD for at least three independent experiments. Data were statistically analyzed using the t-test using GraphPad Prism 8.0. *p < 0.05 was considered statistically significant, ****p < 0.001 was highly statistically significant.

## 3 Result

### 3.1 In silico SwissADME profile and protein target of salidroside

**Figure 1:**
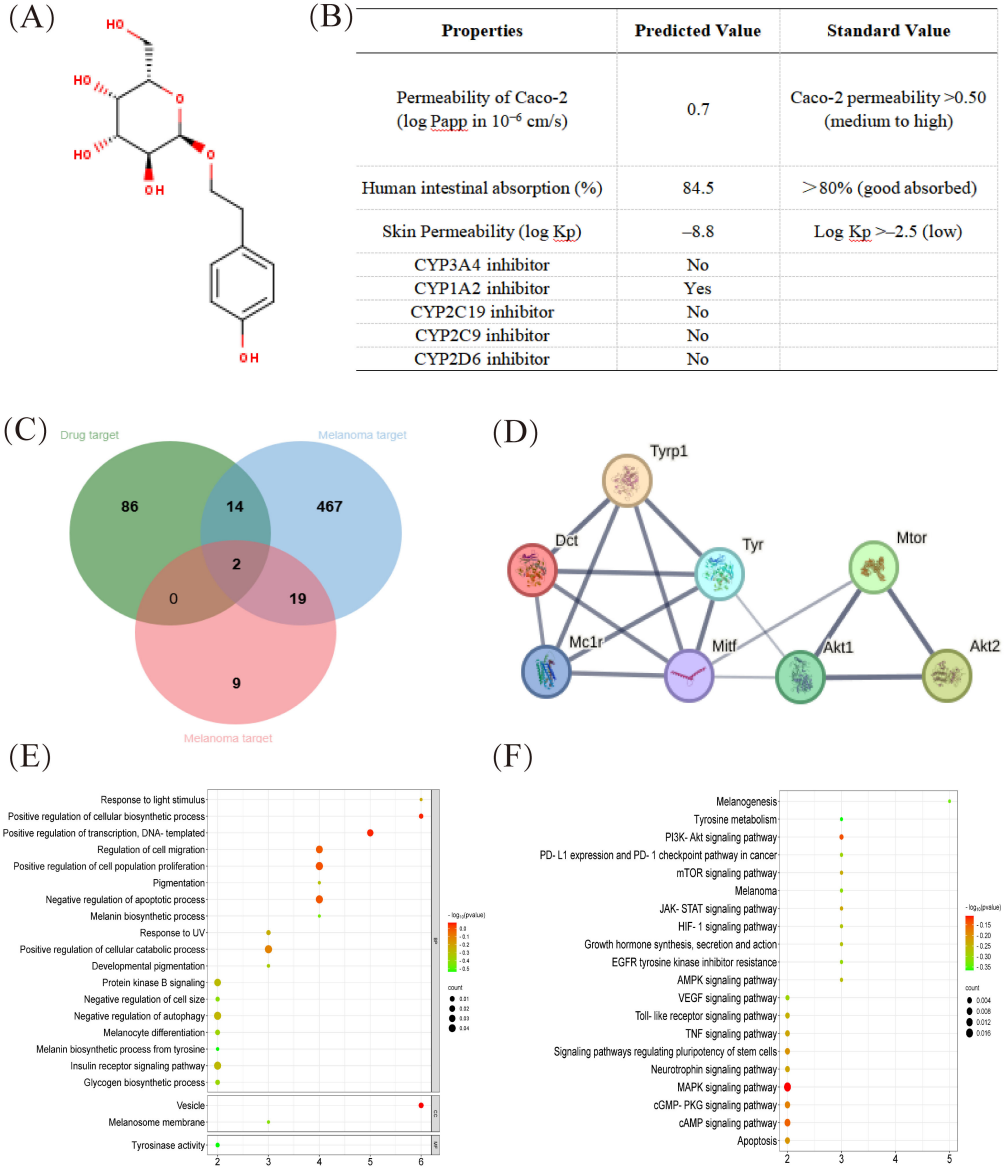
In silico SwissADME profile and protein target of salidroside. **(A)** Chemical structure of salidroside. **(B)** Pharmacokinetic properties of salidroside calculated with the SwissADME database. **(C)** The Venn map related to overlapping genes from bioactive compounds in salidroside and melanoma-related genes. **(D)** The cluster analysis of the central eight targets. The central eight targets were identified as core targets based on degree values. **(E)** GO enrichment analysis (Biological process, molecular function and cellular component enrichment analysis). **(F)** KEGG pathway enrichment analysis. GO, Gene Ontology; KEGG, Kyoto Encyclopedia of Genes and Genomes.

The designing of novel candidates requires substantial attention to their pharmacokinetic properties, including intestinal absorption, skin penetration, and inhibition of CYP isoforms. Based on relevant pharmacokinetic parameters, we determined the molecular formula of salidroside (**Figure 1A**) and its efficacy and safety *in vivo*. As shown in **Figure 1B**, salidroside was predicted to be absorbed in the gastrointestinal (GI) tract and penetrate the stratum corneum, which is the rate-limiting barrier for skin penetration. Additionally, the compound was predicted to inhibit several CYP enzymes, including CYP1A2 and CYP2C19 (**Figure 1B**).

Using disease target databases, 502 potential therapeutic targets for melanoma were predicted from OMIN databases (blue area) and 30 potential therapeutic targets were predicted from Dis Ge NET databases (red area), whereas 102 target proteins were identified as the effective active compounds of salidroside based on the Swiss Target Prediction databases (green area). Among these, two overlapping targets (TYR and MITF) were considered potential targets for the treatment of melanoma. As shown in **Table 3**, fourteen overlapping targets (GSK3B, MAPK1, HSPA8, VEGFA, FGF2, MTOR, and so on) were considered potential targets for the treatment of melanoma with salidroside (**Figure 1C**). A protein–protein interaction (PPI) network was constructed based on eight overlapping targets, consisting of eight nodes and 16 edges. The 8 targets including Tyrosinase-related protein 1 (Tyrp1), Dopachrome tautomerase (Dct), Induced myeloid leukemia cell differentiation protein Mcl-1 homolog (Mcl1), Tyrosinase (Tyr), microphthalmia-associated transcription factor (Mitf), AKT Serine/Threonine Kinase 1 (Akt1), AKT Serine/Threonine Kinase 2 (Akt2), and Mammalian target of rapamycin (Mtor). Cluster analysis revealed that melanin synthesis and cell growth were the primary targets of salidroside in melanoma cells (**Figure 1D**).

**Table 3:** The Venn map related to overlapping genes name.

Number	Gene
1	ADORA3
2	GSK3B
3	MAPK1
4	HSPA8
5	VEGFA
6	FGF2
7	HPSE
8	MMP1
9	ADAM17
10	PRKCB
11	MMP7
12	CDK2
13	EGFR
14	MTOR

Research has found that eumelanin and pheomelanin (two types of melanin), which cause oxidative stress, can also prevent melanoma progression in the early stages (9). A finding showed that MITF, the master regulator of melanocyte differentiation, exhibits an inverse correlation with melanoma cell motility and invasiveness at lower expression levels (32). Gene Ontology (GO) enrichment analysis revealed that salidroside was considerably enriched in inhibiting melanin biosynthesis and pigmentation, regulating nitric oxide biosynthesis, and promoting energy reserve metabolic processes (**Figure 1E**) (23). Kyoto Encyclopedia of Genes and Genomes (KEGG) pathway enrichment analysis identified 95 pathways, and the top 21 enriched pathways (excluding cancer, growth, and immune-related pathways) suggested that salidroside may inhibit melanin synthesis through multiple pathways, such as melanogenesis, tyrosine metabolism, PI3K-Akt, and mTOR signaling pathways (**Figure 1F**).

### 3.2 Salidroside affects melanin in early zebrafish embryos

**Figure 2:**
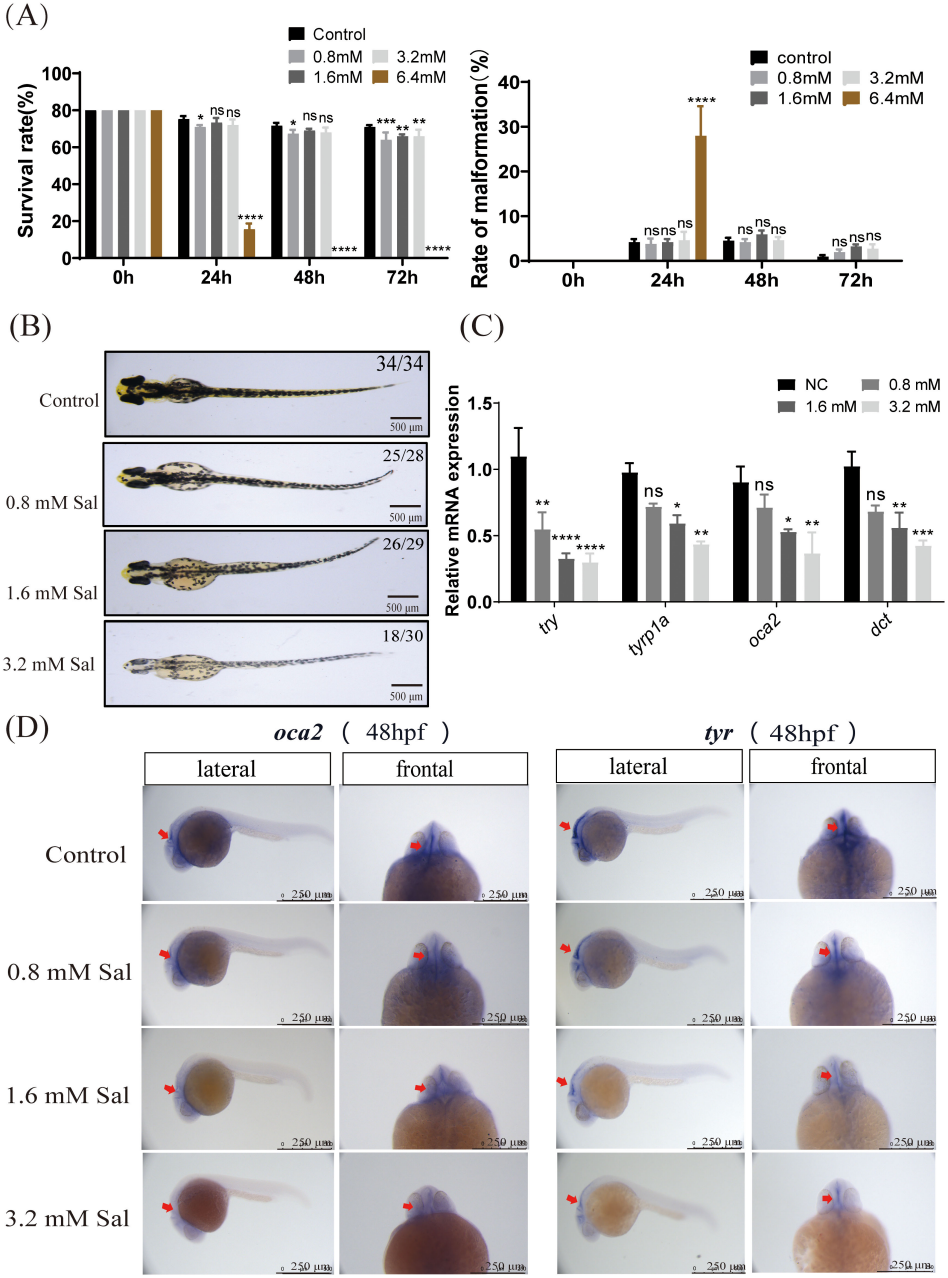
Salidroside affected melanin in early zebrafish embryos. **(A)** Survival rate of embryos within 72-h salidroside-treated zebrafish embryos and morphological deformity rates (including pericardial oedema, spine curvature, and tail abnormality) in salidroside-treated (0–72 hpf) zebrafish embryos (n = 80 for each group). **(B)** Representative images of zebrafish embryos after 48 h of salidroside exposure at different concentrations (0, 0.8,1.6 and 3.2 mM). Scale bar, 500 μm. **(C)** Effects of salidroside on mRNA expression levels of genes related to melanin synthesis in early zebrafish embryos. ns, no significance, *p < 0.05, **p < 0.01, ***p < 0.005, ****p < 0.001 compared with the control. Results were denoted as mean ± SD of three independent experiments. **(D)** Expression patterns of *oca2* and *tyr* by whole-mount *in situ* hybridization in two-day-old zebrafish embryos. Scale bars, 250 μm. SD, standard deviation.

To study the *in vivo* anti-melanin synthesis effects of salidroside, we first selected the appropriate experimental concentration of zebrafish embryos. Below 3.2 mM salidroside did not affect the survival of embryos and the morphological percentage (**Figure 2A**). No viable embryos remained for malformation assessment at 6.4 mM beyond 24h due to complete mortality. Then, we observed that below 3.2 mM salidroside suppressed pigmentation in two-day-old embryos (**Figure 2B**). In addition, Salidroside dose-dependently inhibited mRNA expression of *tyr*, *tyrp1a*, *oca2*, and *dct* in embryos (**Figure 2C**). *In situ* hybridization results showed that salidroside downregulated the expression of *oca2* and *tyr* in two-day-old embryos (**Figure 2D**). Furthermore, previous studies have indicated that antioxidant activity is closely related to melanin synthesis, prompting us to investigate the effects of salidroside on antioxidant enzyme activity in this context (33). We evaluated antioxidant activities by analyzing individual embryos. The data demonstrated that salidroside significantly enhanced SOD, CAT and GSH activities (*p*<0.05, vs control) confirming its antioxidant efficacy (**Supplementary Figure 1**). Taken together, these results showed that the anti-melanin synthesis effect of salidroside may be related to its antioxidant activity.

### 3.3 Salidroside inhibited H_2_O_2_-induced melanogenesis by promoting antioxidant effect in B16F10 cells

To study the *in vitro* anti-melanoma and anti-melanin synthesis effects of salidroside, we examined the effects of salidroside and H_2_O_2_ on melanin synthesis in murine B16F10 melanoma cells using the MTT assay and RT-qPCR. The MTT assay results showed that salidroside had no effect the cell viability and reduced the melanin content of cells in a dose-dependent manner; furthermore, H_2_O_2_ (350 μM, an oxidant used as the negative control) reduced cell viability and increased the melanin content of cells (**Supplementary Figures 2A, B**). And then, the results of tyrosinase activity showed that salidroside considerably inhibited tyrosinase activity at concentrations greater than 40 μM. In addition, the cellular tyrosinase activity was increased by treatment with H_2_O_2_ (**Supplementary Figure 2C**). The mRNA levels of the genes involved in melanin synthesis were reduced, whereas H_2_O_2_ induced an increase (**Supplementary Figure 2D**). Next, we examined the effects of salidroside on melanin synthesis using western blotting. Salidroside downregulated Tyr and Mitf protein expression in cells. In addition, H_2_O_2_ did not upregulate the expression of Tyr protein; however, it upregulated the expression of Mitf protein compared with that in untreated cells (**Supplementary Figure 2E**).

**Figure 3:**
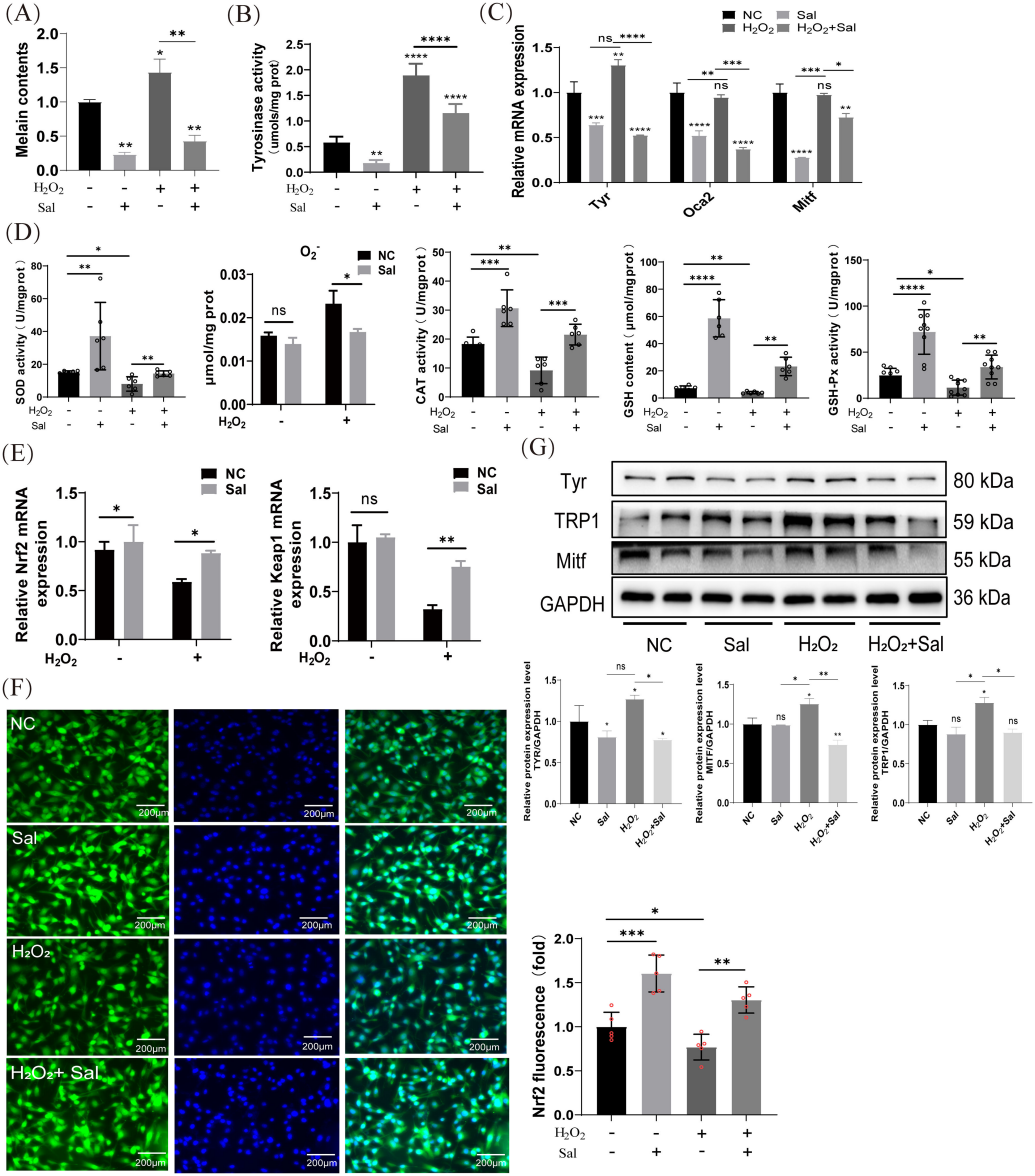
Salidroside inhibits H_2_O_2_-induced melanogenesis by promoting antioxidant effect in B16F10 cells. **(A)** Salidroside treatment for 24 h reduced melanin synthesis after H_2_O_2_ induction. *p < 0.05, **p < 0.01, n=3 **(B)** Cellular tyrosinase activity and tyrosinase activity levels were measured by dopachrome formation from L-DOPA as a substrate. **(C)** The mRNA expression levels of *Tyr*, *Mitf* and *Oca2* in B16F10 cells were measured by RT-qPCR. ns, no significance, *p < 0.05, **p < 0.01, *** *p* < 0.005, ****p < 0.001, n=3. **(D)** B16F10 cells were treated with salidroside for 24 h or H_2_O_2_ for 4 h and cell homogenates were obtained using an ultrasonic crusher. The levels of SOD, O_2_^•−^ content, CAT, GSH and GSH-px in the cells were measured by the corresponding kits. All data are expressed as mean ± SD (n = 6/9). ns, no significance, *p < 0.05, **p < 0.01, ***p < 0.005, ****p < 0.001. **(E)** The mRNA expression levels of *Nrf2* and *Keap1* in B16F10 cells were determined by RT-qPCR. ns, no significance, *p < 0.05, **p < 0.01. **(F)** Immunofluorescence staining results of Nrf2 in B16F10 cells (n=4), scale bar=200 μm. **p < 0.01, ***p < 0.005. **(G)** The protein-expression levels of Tyr, TRP-1, and Mitf were examined by western blotting analysis and greyscale analysis of proteins. GAPDH functioned as a loading control. NC, untreated control; Sal, salidroside; H_2_O_2_ + salidroside, After H_2_O_2_ induction for 4 h ns, no significance, *p < 0.05, **p < 0.01. Results were denoted as mean ± SD of three times. L-DOPA, l-3,4-dihydroxyphenylalanine; SOD, superoxide dismutase; O_2_^•−^, superoxide anion; CAT, catalase; GSH, glutathione; GSH-Px, Glutathione peroxidase; RT-qPCR, quantitative reverse transcription polymerase chain reaction; GAPDH, glyceraldehyde 3-phosphate dehydrogenase; SD, standard deviation.

After determining whether salidroside has anti-melanin synthesis properties, we further explored whether the antioxidant capacity of salidroside plays a role in the cell models. Melanin content analysis showed that salidroside effectively alleviated H_2_O_2_-induced melanin accumulation in B16F10 cells (**Figure 3A**). Salidroside significantly inhibited H_2_O_2_-induced tyrosinase activity in B16F10 cells (**Figure 3B**). The RT-qPCR results showed that salidroside reduced the mRNA expression levels of *Tyr*, *Mitf*, and *Oca2* in both the blank control and H_2_O_2_-induced cells. In contrast, H_2_O_2_ treatment alone increased the expression of these genes (**Figure 3C**). Considering the antioxidant effect of salidroside, we examined the contents of antioxidant enzymes SOD, O_2_^•−^, GSH, GSH-px, and CAT were significantly reduced in H_2_O_2_-induced cells (p < 0.05). However, salidroside significantly reversed these changes (*p* < 0.01) (**Figure 3D**). Additionally, Nrf2 (nuclear factor erythroid 2 related factor) and Keap1 (Kelch-like ECH-associated protein 1) play important roles in antioxidative stress. RT-qPCR results showed that salidroside dramatically increased the mRNA levels of *Nrf2* and *Keap1* in H_2_O_2_-induced cells (**Figure 3E**). Additionally, we observed that under conditions without H_2_O_2_ induction, salidroside upregulated the expression of Nrf2. Activated Nrf2 translocated into the nucleus and initiated the expression of various antioxidant genes, including SOD, catalase, and HO-1. Further analysis by immunofluorescence verified that the expression of Nrf2 in the nucleus increased with stimulation of salidroside, but which could be weakened by H_2_O_2_ (**Figure 3F**, **Supplementary Figure 3**). Activation of Tyrosinase and TRP1 is important in melanin synthesis. Western blotting showed that H_2_O_2_-induced cells promoted TRP-1 and Mitf protein levels, whereas salidroside effectively downregulated the levels of Tyr, TRP-1, and Mitf (**Figure 3G**). In conclusion, salidroside may exert a anti-melanin synthesis effect by inhibiting oxidative stress in B16F10 cells.

### 3.4 Salidroside inhibited H_2_O_2_-inductd melanogenesis by inactivating PI3K/AKT/mTOR

**Figure 4:**
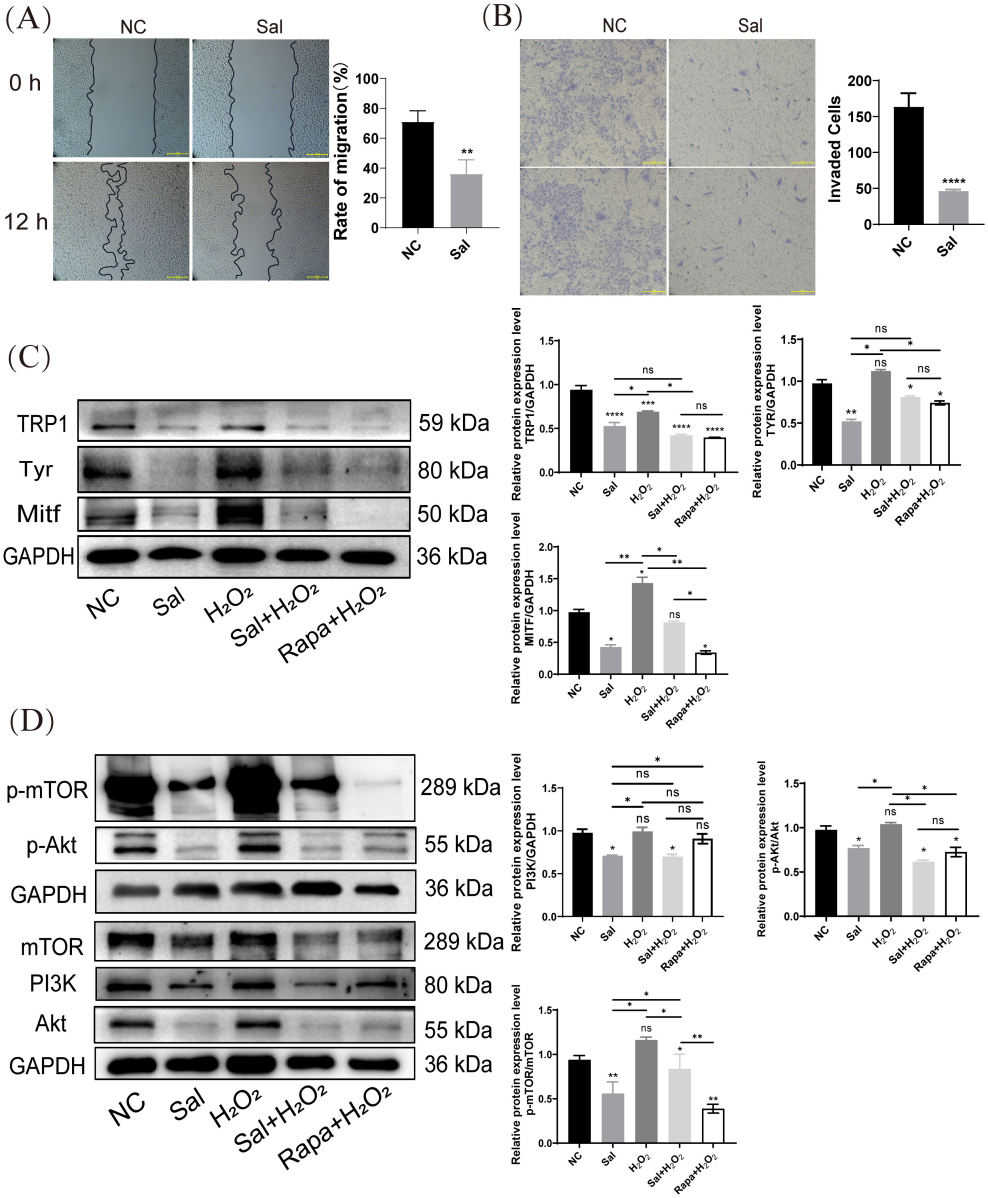
Salidroside inhibits H_2_O_2_-inductd melanogenesis by inactivating PI3K/AKT/mTOR. **(A)** Wounding-healing assays conducted in B16F10 cells. Cells were treated with salidroside (Sal) in serum-free 1640 medium. The wounds were photographed at 0 and 12 h after salidroside treatment. Representative photos from each group are presented in the left panels, migration rates are shown in the right panels. Data are shown as mean ± SD of three independent experiments, **p < 0.01, n = 3, scale bar = 100 μm. **(B)** After 24 h of salidroside treatment, B16F10 cells was detected by Trans-well assay. ****p < 0.001, n = 3, scale bar = 100 μm. **(C)** The protein-expression levels of melanin synthesis-related proteins (TRP1, Tyr and Mitf) in B16F10 cells were detected by western blotting. **(D)** The protein-expression levels of PI3K/Akt/mTOR signaling pathway-related proteins (PI3K, Akt, and mTOR) in B16F10 cells were detected by western blotting. GAPDH functioned as a loading control. NC: untreated control; Sal: salidroside; H_2_O_2_ + salidroside: After H_2_O_2_ induction for 4 h, incubation with salidroside (100 μM) was performed for 24 h; H_2_O_2_ + Rapa: After H_2_O_2_ induction for 4 h, incubation with rapamycin (2 μM) was performed for 24 (h) ns, no significance, *p < 0.05, **p < 0.01, vs. control group. Results are denoted as mean ± SD. GAPDH, glyceraldehyde 3-phosphate dehydrogenase; SD, standard deviation.

Given that salidroside inhibited melanin synthesis in H_2_O_2_-inducted cells, we explored whether salidroside inhibits B16F10 cell migration. The results of the wound-healing and transwell assays reflected the effects of salidroside on melanoma cell migration. As shown in **Figure 4A**, B16F10 cells exhibited a migratory tendency after 12 h, whereas salidroside treatment inhibited cell migration (**Figures 4A, B**). Several studies claimed that salidroside inactivated the mTOR pathway (34, 35). Combined with disease and drug target databases, we found that salidroside may modulate the PI3K-Akt signaling pathway by targeting mTOR, thus exerting a therapeutic effect on melanoma. Therefore, we used Rapamycin (rapa, 2 μM, an mTOR inhibitor) as the positive control. Western blotting analysis showed that rapa reduced melanin synthesis in H_2_O_2_-induced cells (**Figure 4C**). Moreover, both salidroside and rapa downregulated the expression of PI3K, phospho-Akt (Ser473), and phospho-mTOR (Ser2481) in B16F10 cells, whereas H_2_O_2_ did not affect their levels. Salidroside exerted effects comparable to those of rapa (2 μM) (**Figure 4D**).

### 3.5 Salidroside suppressed tumor growth in a syngeneic tumor model

**Figure 5:**
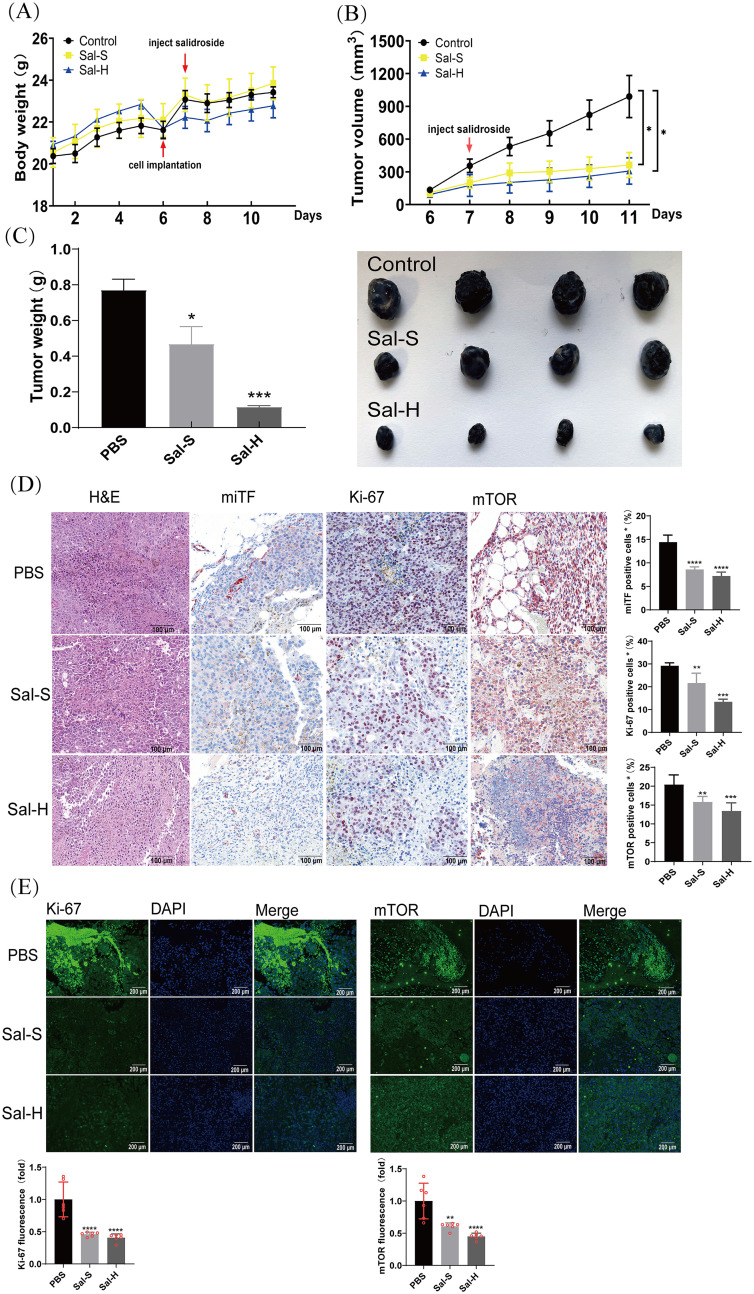
Salidroside suppresses tumor growth in syngeneic tumor model. **(A)** Body weight of mice during the treatment period. **(B)** Tumor volume of mice during the treatment period. *p < 0.05. **(C)** Tumor weight (left panel) and representative photos of tumor dissected from mice (right panel). *p < 0.05, ***p < 0.005, vs. control group. n=4. Sal-S: salidroside 4 mg/kg. Sal-H: salidroside 8 mg/kg. **(D)** HE and IHC staining of Ki-67, miTF, and mTOR in tumor tissues. Representative melanoma sections from mice of each group are presented in the left panels. Relative positive rates are shown in the right panels. Data are presented as mean ± SD of four mice. **p < 0.01, ***p < 0.005, ****p < 0.001. vs. control group, scale bar = 100 μm. **(E)** The expression of Ki-67 and mTOR were detected by IHC assay in the tumor issues of mice (n=4). Data were presented as the mean ± SD, scale bar = 200 μm, **p < 0.01, ****p < 0.001. HE, hematoxylin and eosin; IHC, immunohistochemistry; SD, standard deviation.

To determine the anti-melanoma and anti-melanin synthesis effects of salidroside *in vivo*, we established a B16F10 melanoma mouse model. Four-week-old BALB/c male mice, weighing approximately 20 g, were randomized into three groups: vehicle control, 4 mg/kg salidroside, and 8 mg/kg salidroside. When the tumor diameter reached 300 mm^3^, the treatment was initiated. Salidroside was injected intratumorally for 5 days. As shown in the results, salidroside and the vehicle control had no significant influence on mouse weight (**Figure 5A**). In addition, we also discovered no significant changes in serum ASL/ALT and creatinine levels (**Supplementary Figure 4A**). which indicating that salidroside at 4 and 8 mg/kg is safe for intratumoral administration. Salidroside at 8 mg/kg showed the strongest anti-tumor effects. Salidroside (8 mg/kg) decreased the average tumor weight and volume (**Figures 5B, C**). Previous results have demonstrated that salidroside exhibits antioxidant properties, which can influence melanin formation. Therefore, we further investigated the changes in antioxidant enzyme levels in mice from the treatment group. The results showed that, following salidroside injection, the activities of superoxide dismutase (SOD) and catalase (CAT) in mouse serum significantly increased, indicating that salidroside enhanced the antioxidant capacity *in vivo* (**Supplementary Figure 4B**).

To investigate the potential biological mechanisms of salidroside in melanoma growth, we discovered clearly inhibited melanoma-related angiogenesis by H&E staining. Additionally, miTF acts a master regulator in melanoma and Ki-67 plays a proliferation marker, reflecting tumor aggressiveness and predicting prognosis (36). We detected miTF and Ki-67 in melanoma tissues and found that the miTF and Ki-67 were downregulated in the salidroside-treated group. After determining that salidroside downregulated the mTOR levels in B16F10 cells, we further examined the effects of salidroside on mTOR expression in melanoma tissues. The obtained results showed that salidroside decreased the percentage of mTOR-positive B16F10 melanoma cells (**Figure 5D**). We further investigate the cellular localization and dynamic changes of these markers by immunofluorescence. We found that both concentrations of salidroside could inhibit the expression of Ki-67 and mTOR in mouse melanoma (**Figure 5E**). Overall, the results of the animal experiments indicated that salidroside exerts anti-melanoma growth effects *in vivo*.

## 4 Discussion

Melanoma is a type of skin cancer that originates from pigment-producing cells known as melanocytes. Melanocytes produce melanin via melanogenesis (37). In 2020, the National Cancer Institute released a report stating that although the risk of developing melanoma tends to increase with age, it can occur in any age group (38). The reduction in melanoma progression depends on many factors, such as ratio of melanin and oxidative stress (9). Some studies suggest that oxidative stress impairs cancer cell survival during the early stages of melanoma development and growth (39). In addition, melanin acts as a double-edged sword in melanoma: while offering antioxidant protection by scavenging reactive oxygen species, it also shields tumor cells from oxidative damage, contributing to therapy resistance. Photodynamic Therapy (PDT) is an emerging complementary and unconventional treatment for melanoma. Although PDT can treat melanoma, melanoma defense systems (such as pigmentation and antioxidative stress) are major factors that reduce the efficacy of PDT (40). Uncontrolled melanogenesis plays a role in the progression of melanotic melanoma, and melanin pigments can reduce the efficacy of radiotherapy and immunotherapy (41), indicating that increased melanogenesis correlates with heightened melanoma aggressiveness.

In light of evidence highlighting the importance of melanin in regulating melanoma development, it is vital to recognize the possibility of using compounds or natural products that decrease melanin synthesis as potential treatment options to prevent melanoma progression. Certain natural products, compounds, and drugs can stimulate melanin synthesis by interfering with the melanogenesis signaling pathways. Huey-Chun Huang et al. revealed that theophylline promotes melanogenesis in B16F10 cells by upregulating the mitogen-activated protein kinase 1 (MEK1/2) and Wnt/β-catenin signaling pathways (42). Tabolacci et al. focused on the potential ability of Aloe-emodin (AE) to reduce melanoma cell proliferation and metastatic potential by inducing cell differentiation. They demonstrated the inhibitory effects of AE on melanoma cell proliferation and invasion, accompanied by stimulation of cell-differentiation parameters (43). In summary, melanogenesis hinder melanoma treatment while promoting the melanoma progression.

A detailed review revealed that salidroside extracts and compounds display numerous bioactivities, including antioxidative, anti-aging, anti-cancer, and neuroprotective activities, and possess crucial adaptogenic properties (44). Amount of evidence showed that SOD, GSH, and GSH-px were the representative makers associated with oxidative stress (45). Obviously, the level of SOD, GSH, and GSH-px were increased in H_2_O_2_-inducted models or melanoma model, whereas salidroside treatment, significantly improved SOD, GSH, and GSH-px expression and increased the expression of Nrf2 in nucleus. Our results suggested that the anti- melanogenesis effect of salidroside by inhibited oxidative stress.

Signaling pathways involve a network of proteins and enzymes that regulate melanocyte function and melanoma progression. For example, the cAMP/PKA and MAPK pathways are central regulators of melanin synthesis and melanoma development (46). We also found that Nrf2 is not only the downstream gene of PI3K/Akt but also an important transcriptional activator in the cytoplasm (47). Importantly, by mapping the effects of salidroside on these pathways by cluster analysis, we identified critical target molecules (AKT, mTOR, and MITF) that were directly affected by the compound, which may serve as biomarkers for assessing salidroside against melanoma growth and potential drug development. Kim et al. concluded that melanogenic programs can crosstalk with the signaling pathways of NF-κB activation through toll-like receptors (TLRs) (48, 49). We elucidated that H_2_O_2_ promotes mTOR expression and upregulates the PI3K/Akt signaling pathway to activate the downstream targets, Tyr and Mitf. In contrast, salidroside inhibited mTOR, Tyr, and Mitf expression. Importantly, through the validation of rapamycin, mTOR was found to be a target for salidroside inhibition of melanin formation. In addition, we demonstrated that salidroside can effectively inhibit tumor formation by intratumoral administration. This mode of administration maximizes drug concentrations at the tumor site while minimizing systemic toxicity – demonstrating the innovative potential of this delivery method. Taken together, our findings indicate that salidroside regulates PI3K/Akt/mTOR expression and inhibits melanin formation.

To the best of our knowledge, the present study is the first to validate the pharmacological effects of salidroside on melanoma networks using both *in vitro* and *in vivo* experiments. It is also the first to identify TYR and MITF as key protein targets of salidroside in melanoma. Melanogenesis, tyrosine metabolism, and PI3K-AKT signaling pathways play important roles in the treatment of melanoma, according to KEGG analysis. These experiments demonstrate that salidroside reflects a significant anti-melanogenic effect by inhibiting oxidative stress *in vitro* and *in vivo*. From a mechanistic perspective, western blot results showed that H2O2 significantly increased the phosphorylation of mTOR and Akt, and this phenomenon can be reversed by salidroside. Simultaneously, salidroside enhanced Nrf2 translocation to the nucleus suggesting that salidroside inhibits melanogenesis by inhibiting oxidative stress. Salidroside’s multi-target regulatory capacity, low toxicity, and efficacy make it a promising candidate for developing an effective anti-melanoma therapeutic agent.

## 5 Conclusion

Network pharmacology was therefore determined as an effective method for delineating the therapeutic effects of traditional herbs on different diseases. In conclusion, our results confirmed the network pharmacology results showing that salidroside has anti-melanoma effects in mouse models. We found that the anti-melanoma effect of salidroside by inhibiting oxidative stress and melanin synthesis. These results indicated that salidroside has the potential to be developed as a novel drug for the treatment of melanoma. Moreover, the present study findings confirmed that the inhibition of PI3K/Akt signaling by directly targeting mTOR is a feasible strategy for melanoma management. CurrenTtly, research on salidroside is limited to animal experiments and is expected to continue in human cell lines or clinical research to apply these findings to human melanoma.

## Data Availability

The original contributions presented in the study are included in the article/**Supplementary Material**. Further inquiries can be directed to the corresponding authors.

## References

[1] Siegel RL Miller KD Jemal A. Cancer statistic. CA Cancer J Clin. (2020) 70:7–30. doi: 10.3322/caac.21590, PMID: 31912902

[2] Gershenwald JE Scolyer RA Hess KR Sondak VK Long GV Ross MI. Melanoma staging: Evidence-based changes in the American Joint Committee on Cancer eighth edition cancer staging manual. CA Cancer J Clin. (2017) 67:472–92. doi: 10.3322/caac.21409, PMID: 29028110 PMC5978683

[3] Emanuelli M Sartini D Molinelli E Campagna R Pozzi V Salvolini E. The Double-Edged Sword of Oxidative Stress in Skin Damage and Melanoma: From Physiopathology to Therapeutical Approaches. Antioxidants (Basel). (2022) 11(4):612. doi: 10.3390/antiox11040612, PMID: 35453297 PMC9027913

[4] Slominski RM Kim T-K Janjetovic Z Brożyna AA Podgorska E Dixon KM. Malignant Melanoma: An Overview, New Perspectives, and Vitamin D Signaling. Cancers. (2024) 16:2262. doi: 10.3390/cancers16122262, PMID: 38927967 PMC11201527

[5] Brożyna AA Jóźwicki W Roszkowski K Filipiak J Slominski AT. Melanin content in melanoma metastases affects the outcome of radiotherapy. Oncotarget. (2016) 7:17844–53. doi: 10.18632/oncotarget.7528, PMID: 26910282 PMC4951254

[6] Major E Lin KH Lee SC Káldi K Győrffy B Tigyi GJ. LPA suppresses HLA-DR expression in human melanoma cells: a potential immune escape mechanism involving LPAR1 and DR6-mediated release of IL-10. Acta Pharmacol Sin. (2025) 46:222–30. doi: 10.1038/s41401-024-01373-x, PMID: 39187677 PMC11696067

[7] Lee A Park H Lim S Lim J Koh J Jeon YK. Novel role of microphthalmia-associated transcription factor in modulating the differentiation and immunosuppressive functions of myeloid-derived suppressor cells. J Immunother Cancer. (2023) 11:e005699. doi: 10.1136/jitc-2022-005699, PMID: 36627143 PMC9835954

[8] Cabaço LC Tomás A Pojo M Barral DC. The Dark Side of Melanin Secretion in Cutaneous Melanoma Aggressiveness. Front Oncol. (2022) 12:887366. doi: 10.3389/fonc.2022.887366, PMID: 35619912 PMC9128548

[9] Saud A Sagineedu SR Ng HS Stanslas J Lim JCW. Melanoma metastasis: What role does melanin play? (Review). Oncol Rep. (2022) 48:217. doi: 10.3892/or.2022.8432, PMID: 36281942

[10] Slominski A Paus R Mihm MC. Inhibition of melanogenesis as an adjuvant strategy in the treatment of melanotic melanomas: selective review and hypothesis. Anticancer Res. (1998) 18 (5B):3709–15., PMID: 9854482

[11] Sinha S Singh SK Jangde N Ray R Rai V. p32 promotes melanoma progression and metastasis by targeting EMT markers, Akt/PKB pathway, and tumor microenvironment. Cell Death Dis. (2021) 12:1012. doi: 10.1038/s41419-021-04311-5, PMID: 34711805 PMC8553772

[12] Denecker G Vandamme N Akay O Koludrovic D Taminau J Lemeire K. Identification of a ZEB2-MITF-ZEB1 transcriptional network that controls melanogenesis and melanoma progression. Cell Death Differ. (2014) 21:1250–61. doi: 10.1038/cdd.2014.44, PMID: 24769727 PMC4085532

[13] Zhang X Xie L Long J Xie Q Zheng Y Liu K. Salidroside: A review of its recent advances in synthetic pathways and pharmacological properties. Chem Biol Interact. (2021) 339:109268. doi: 10.1016/j.cbi.2020.109268, PMID: 33617801

[14] Panossian A Wikman G Sarris J. Rosenroot (Rhodiola rosea): traditional use, chemical composition, pharmacology and clinical efficacy. Phytomedicine. (2010) 17:481–93. doi: 10.1016/j.phymed.2010.02.002, PMID: 20378318

[15] Morikawa T. Bio-Functional Natural Products in Edible Resources for Human Health and Beauty. Molecules. (2022) 27(16):5060., PMID: 36014313 10.3390/molecules27165060 PMC9413208

[16] Alharbi NM Alhashim HM. Beauty Salons are Key Potential Sources of Disease Spread. Infect Drug Resist. (2021) 14:1247–53. doi: 10.2147/IDR.S303461, PMID: 33790595 PMC8007475

[17] Yang S Wang L Zeng Y Wang Y Pei T Xie Z. Salidroside alleviates cognitive impairment by inhibiting ferroptosis via activation of the Nrf2/GPX4 axis in SAMP8 mice. Phytomedicine. (2023) 114:154762. doi: 10.1016/j.phymed.2023.154762, PMID: 36965372

[18] Snyman M Walsdorf RE Wix SN Gill JG. The metabolism of melanin synthesis-From melanocytes to melanoma. Pigment Cell Melanoma Res. (2024) 37:438–52. doi: 10.1111/pcmr.13165, PMID: 38445351 PMC11178461

[19] Slominski A Tobin DJ Shibahara S Wortsman J. Melanin pigmentation in mammalian skin and its hormonal regulation. Physiol Rev. (2004) 84:1155–228. doi: 10.1152/physrev.00044.2003, PMID: 15383650

[20] Zang D Niu C Lu X Aisa HA. A Novel Furocoumarin Derivative, 5-((diethylamino)me-13 thyl)-3-phenyl-7H-furo [3,2-g] chromen-7-one Upregulates Melanin Synthesis via the Activation of cAMP/PKA and MAPKs Signal Pathway: *In Vitro* and *In Vivo* Study. Int J Mol Sci. (2022) 23(22):14190. doi: 10.3390/ijms232214190, PMID: 36430668 PMC9694462

[21] Ren L Bao D Wang L Xu Q Xu Y Shi Z. Nucleobindin-2/nesfatin-1 enhances the cell proliferation, migration, invasion and epithelial-mesenchymal transition in gastric carcinoma. J Cell Mol Med. (2022) 26:4986–94. doi: 10.1111/jcmm.17522, PMID: 36065769 PMC9549493

[22] Man J Zhou W Zuo S Zhao X Wang Q Ma H. TANGO1 interacts with NRTN to promote hepatocellular carcinoma progression by regulating the PI3K/AKT/mTOR signaling pathway. Biochem Pharmacol. (2023) 213:115615. doi: 10.1016/j.bcp.2023.115615, PMID: 37211171

[23] Tang Q-Q Wang Z-D An X-H Zhou X-Y Zhang R-Z Zhan X. Apigenin Ameliorates H2O2-Induced Oxidative Damage in Melanocytes through Nuclear Factor-E2-Related Factor 2 (Nrf2) and Phosphatidylinositol 3-Kinase (PI3K)/Protein Kinase B (Akt)/Mammalian Target of Rapamycin (mTOR) Pathways and Reducing the Generation of Reactive Oxygen Species (ROS) in Zebrafish. Pharmaceuticals. (2024) 17:1302. doi: 10.3390/ph17101302, PMID: 39458943 PMC11510047

[24] Ozeki H Ito S Wakamatsu K Hirobe T. Chemical characterization of hair melanins in various coat-color mutants of mice. J Invest Dermatol. (1995) 105:361–6. doi: 10.1111/1523-1747.ep12320792, PMID: 7665913

[25] Luo L Jia W Zhang Y Guo Y Zhu J Li C. Proprotein Convertase Furin Regulates Melanogenesis via the Notch Signaling Pathway. Discov Med. (2023) 35:144–56. doi: 10.24976/Discov.Med.202335175.15, PMID: 37105924

[26] Chen J Ye W. Molecular mechanisms underlying Tao-Hong-Si-Wu decoction treating hyperpigmentation based on network pharmacology, Mendelian randomization analysis, and experimental verification. Pharm Biol. (2024) 62:296–313. doi: 10.1080/13880209.2024.2330609, PMID: 38555860 PMC11632782

[27] Miricescu D Totan A Stanescu-Spinu II Badoiu SC Stefani C Greabu M. PI3K/AKT/mTOR Signaling Pathway in Breast Cancer: From Molecular Landscape to Clinical Aspects. Int J Mol Sci. (2020) 22(1):173. doi: 10.3390/ijms22010173, PMID: 33375317 PMC7796017

[28] Yu G Nanding A. Salidroside overcomes cisplatin resistance in ovarian cancer via the inhibition of CRNDE-mediated autophagy. Mol Cell Biochem. (2025) 480:3097–116. doi: 10.1007/s11010-024-05168-w, PMID: 39636431

[29] Westerfield M. The zebrafish book: a guide for the laboratory use of zebrafish Danio (Brachydanio) rerio. Eugene: University of Oregon Press (1994).

[30] Chen WY John JA Lin CH Lin HF Wu SC Lin CH. Expression of metallothionein gene during embryonic and early larval development in zebrafish. Aquat Toxicol. (2004) 69:215–27. doi: 10.1016/j.aquatox.2004.05.004, PMID: 15276328

[31] Daina A Michielin O Zoete V. SwissADME: a free web tool to evaluate pharmacokinetics, drug-likeness and medicinal chemistry friendliness of small molecules. Sci Rep. (2017) 7:42717. doi: 10.1038/srep42717, PMID: 28256516 PMC5335600

[32] Hsiao JJ Fisher DE. The roles of microphthalmia-associated transcription factor and pigmentation in melanoma. Arch Biochem Biophys. (2014) 563:28–34. doi: 10.1016/j.abb.2014.07.019, PMID: 25111671 PMC4336945

[33] Hseu YC Vudhya Gowrisankar Y Wang LW Zhang YZ Chen XZ Huang PJ. The *in vitro* and *in vivo* depigmenting activity of pterostilbene through induction of autophagy in melanocytes and inhibition of UVA-irradiated α-MSH in keratinocytes via Nrf2-mediated antioxidant pathways. Redox Biol. (2021) 44:102007. doi: 10.1016/j.redox.2021.102007, PMID: 34049220 PMC8167190

[34] Fan XJ Wang Y Wang L Zhu M. Salidroside induces apoptosis and autophagy in human colorectal cancer cells through inhibition of PI3K/Akt/mTOR pathway. Oncol Rep. (2016) 36:3559–67. doi: 10.3892/or.2016.5138, PMID: 27748934

[35] Rong L Li Z Leng X Li H Ma Y Chen Y. Salidroside induces apoptosis and protective autophagy in human gastric cancer AGS cells through the PI3K/Akt/mTOR pathway. BioMed Pharmacother. (2020) 122:109726. doi: 10.1016/j.biopha.2019.109726, PMID: 31918283

[36] Londhe S Tripathy S Saha S Patel A Chandra Y Patra CR. Therapeutic Potential of Silver Nitroprusside Nanoparticles for Melanoma. ACS Appl Bio Mater. (2024) 7:5057–75. doi: 10.1021/acsabm.4c00597, PMID: 39115261

[37] Ali Z Yousaf N Larkin J. Melanoma epidemiology, biology and prognosis. EJC Suppl. (2013) 11:81–91. doi: 10.1016/j.ejcsup.2013.07.012, PMID: 26217116 PMC4041476

[38] Sung H Ferlay J Siegel RL Laversanne M Soerjomataram I Jemal A. Global Cancer Statistics 2020: GLOBOCAN Estimates of Incidence and Mortality Worldwide for 36 Cancers in 185 Countries. CA Cancer J Clin. (2021) 71:209–49. doi: 10.3322/caac.21660, PMID: 33538338

[39] Aurora AB Khivansara V Leach A Gill JG Martin-Sandoval M Yang C. Loss of glucose 6-phosphate dehydrogenase function increases oxidative stress and glutaminolysis in metastasizing melanoma cells. Proc Natl Acad Sci U.S.A. (2022) 119(6):e2120617119. doi: 10.1073/pnas.2120617119, PMID: 35110412 PMC8833200

[40] Domingues B Lopes JM Soares P Pópulo H. Melanoma treatment in review. Immunotargets Ther. (2018) 7:35–49. doi: 10.2147/ITT.S134842, PMID: 29922629 PMC5995433

[41] Mitra D Luo X Morgan A Wang J Hoang MP Lo J. An ultraviolet-radiation-independent pathway to melanoma carcinogenesis in the red hair/fair skin background. Nature. (2012) 491:449–53. doi: 10.1038/nature11624, PMID: 23123854 PMC3521494

[42] Huang HC Yen H Lu JY Chang TM Hii CH. Theophylline enhances melanogenesis in B16F10 murine melanoma cells through the activation of the MEK 1/2, and Wnt/β-catenin signaling pathways. Food Chem Toxicol. (2020) 137:111165. doi: 10.1016/j.fct.2020.111165, PMID: 32001318

[43] Tabolacci C Lentini A Mattioli P Provenzano B Oliverio S Carlomosti F. Antitumor properties of aloe-emodin and induction of transglutaminase 2 activity in B16-F10 melanoma cells. Life Sci. (2010) 87:316–24. doi: 10.1016/j.lfs.2010.07.003, PMID: 20624404

[44] Zhuang W Yue L Dang X Chen F Gong Y Lin X. Rosenroot (Rhodiola): Potential Applications in Aging-related Diseases. Aging Dis. (2019) 10:134–46. doi: 10.14336/AD.2018.0511, PMID: 30705774 PMC6345333

[45] Yu CC Du YJ Li J Li Y Wang L Kong LH. Neuroprotective Mechanisms of Puerarin in Central Nervous System Diseases: Update. Aging Dis. (2022) 13:1092–105. doi: 10.14336/AD.2021.1205, PMID: 35855345 PMC9286922

[46] Kim SH Lee J Jung J Kim GH Yun CY Jung SH. Interruption of p38(MAPK)-MSK1-CREB-MITF-M pathway to prevent hyperpigmentation in the skin. Int J Biol Sci. (2024) 20:1688–704. doi: 10.7150/ijbs.93120, PMID: 38481807 PMC10929196

[47] Zhang Q Yao M Qi J Song R Wang L Li J. Puerarin inhibited oxidative stress and alleviated cerebral ischemia-reperfusion injury through PI3K/Akt/Nrf2 signaling pathway. Front Pharmacol. (2023) 14:1134380. doi: 10.3389/fphar.2023.1134380, PMID: 37284311 PMC10240043

[48] Boggu P Venkateswararao E Manickam M Kim Y Jung SH. Exploration of SAR for novel 2-benzylbenzimidazole analogs as inhibitor of transcription factor NF-κB. Arch Pharm Res. (2017) 40:469–79. doi: 10.1007/s12272-017-0886-1, PMID: 28108939

[49] Xu YR Lei CQ. TAK1-TABs Complex: A Central Signalosome in Inflammatory Responses. Front Immunol. (2020) 11:608976. doi: 10.3389/fimmu.2020.608976, PMID: 33469458 PMC7813674

