# Supplementary material for: Salidroside inhibits melanin synthesis and melanoma growth via mTOR and PI3K/Akt pathways
Source: Front Oncol. 2025 Jul 10;15:1583580. doi: 10.3389/fonc.2025.1583580 (PMC12287071; doi:10.3389/fonc.2025.1583580)
Supplement: Supplementary file 1 [file DataSheet1.docx]

**SUPPLEMENTARY FIGURES**

**Supplementary Fig. S1**


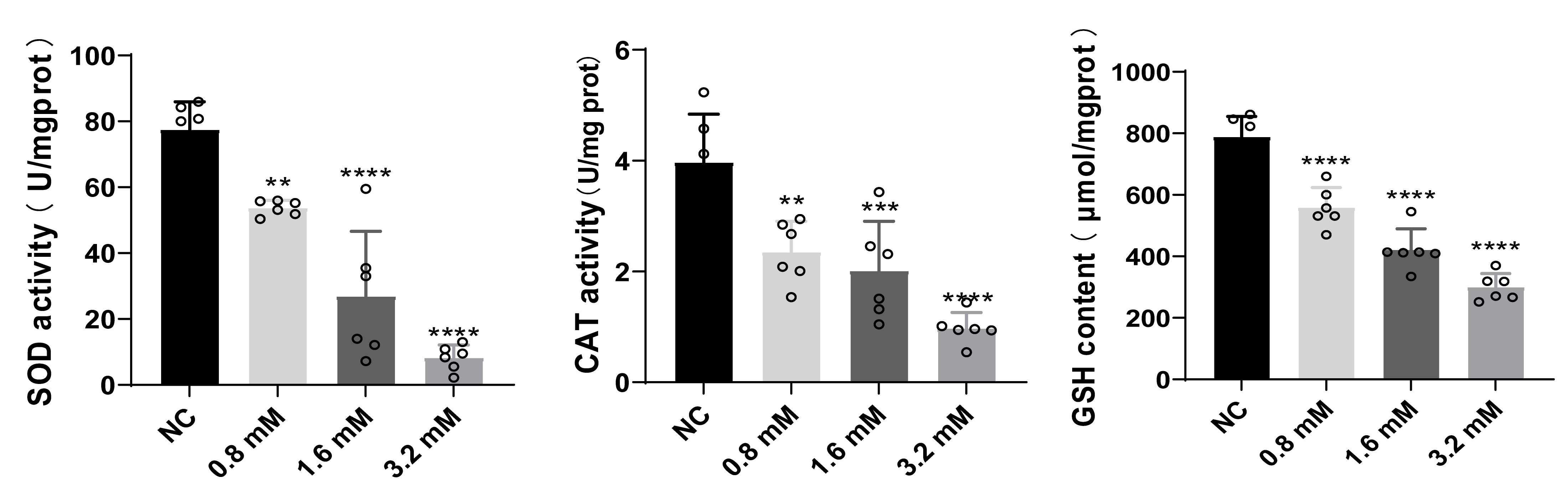


Supplementary Figure S1 Salidroside regulated the oxidative stress level in zebrafish embryos.

Zebrafish embryos were treated with different concentrations salidroside (0, 0.8, 1.6, 3.2 mM) for 48 h. The levels of SOD, CAT and GSH in the embryos were measured by the corresponding kits. All data are expressed as mean ± SD (n = 6). **p* < 0.05, ***p* < 0.01, ****p* < 0.005, *****p* < 0.001.

SOD: Superoxide dismutase; CAT: CAT: catalase; GSH: glutathione.

**Supplementary Fig. S2**


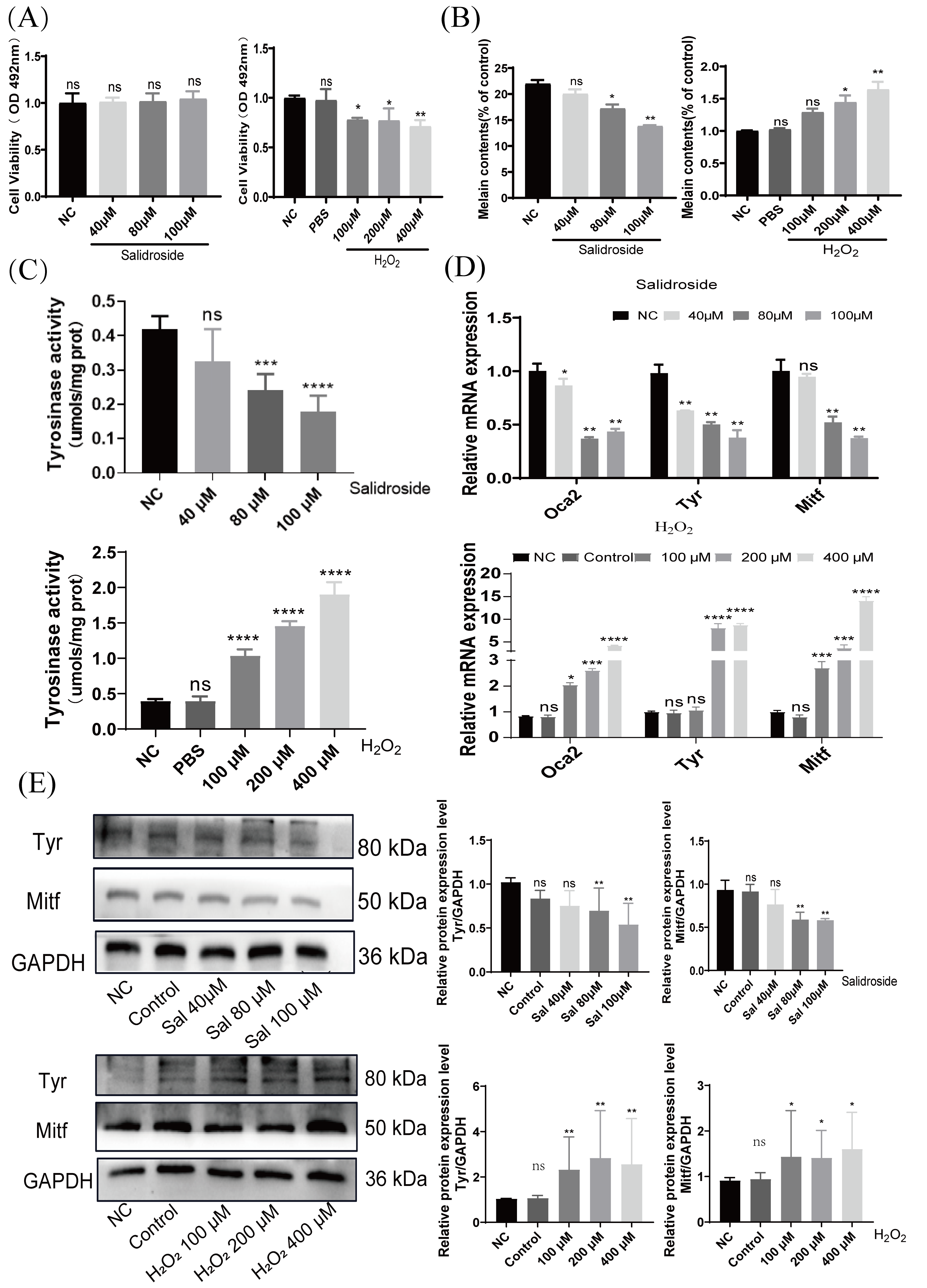


Supplementary Figure S2 Salidroside affects melanin formation in B16F10 cells.

(A) MTT assays in B16F10 cells. Cells were treated with indicated concentrations of salidroside (0-100 μM) for 24 h or H_2_O_2_ (0-400 μM) for 4 h. ns, no significance, **p* < 0.05, ***p* < 0.01. (B) Salidroside or H_2_O_2_ treatment for 24 h reduced melanin synthesis. **p* < 0.05, ***p* < 0.01, n=3. (C) Cellular tyrosinase activity and tyrosinase activity levels were measured by dopachrome formation from L-DOPA as a substrate. ns: no significance, ****p* < 0.005, *****p* < 0.01. (D) The mRNA expression of *Tyr*, *Mitf* and *Oca2* in B16F10 cells were measured by RT-qPCR. ns, no significance, **p* < 0.05, ***p* < 0.01, ****p* < 0.005, *****p* < 0.01. (E) The protein-expression levels of Tyr, TRP-1, and Mitf were examined by western blotting analysis and greyscale analysis of proteins. GAPDH functioned as a loading control. **p* < 0.05, ***p* < 0.01.

L-DOPA: l-3,4-dihydroxyphenylalanine; H_2_O_2_: Hydrogen peroxide; SOD: superoxide dismutase; RT-qPCR: quantitative reverse transcription polymerase chain reaction; GAPDH: glyceraldehyde 3-phosphate dehydrogenase; SD: standard deviation; Tyr: Tyrosine; Mitf: Melanocyte inducing transcription factor; Oca2: OCA2 melanosomal transmembrane protein

**Supplementary Fig. S3**


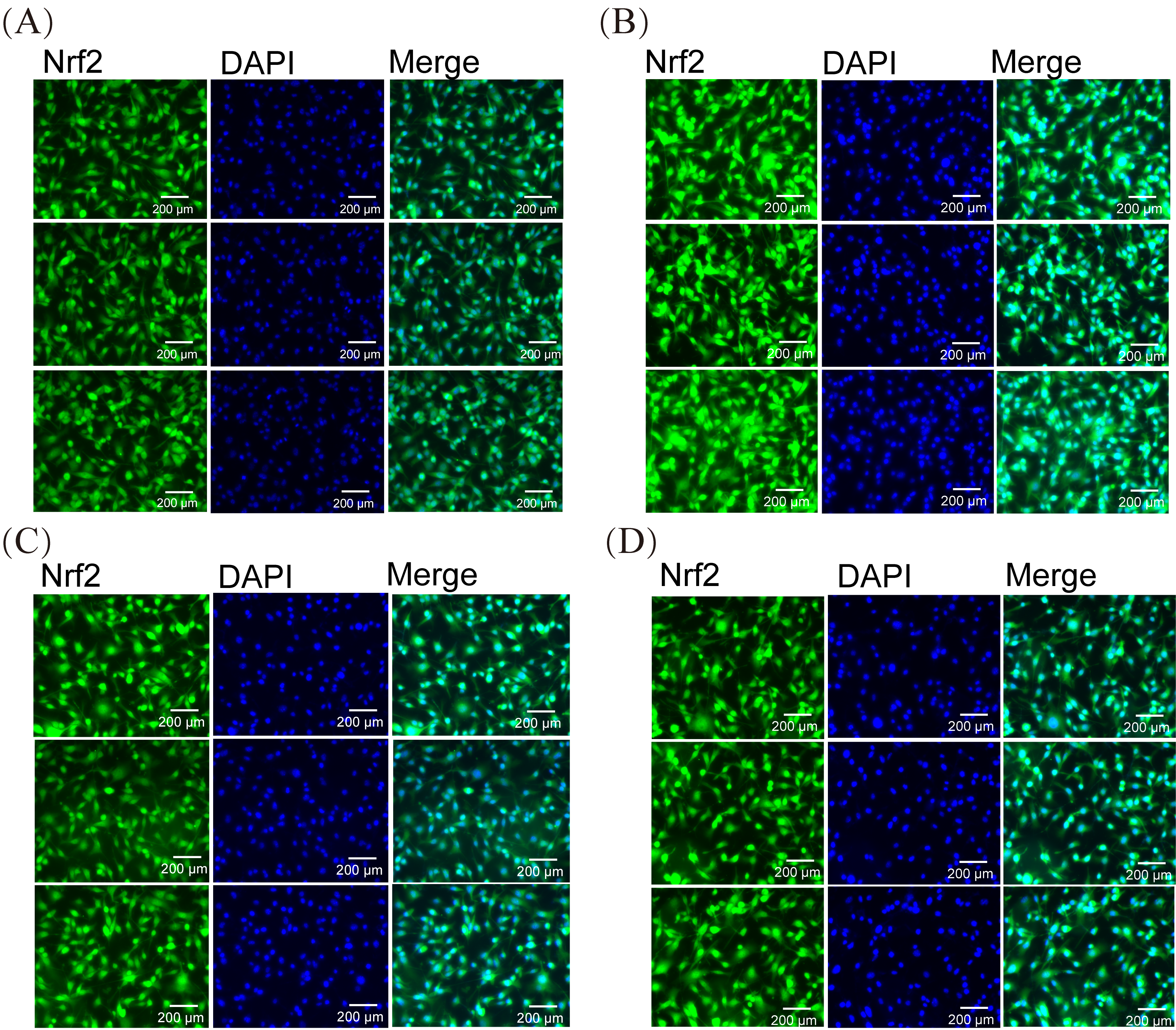


Supplementary Figure 3 Immunofluorescence staining results of Nrf2 in B16F10 cells, which treated with PBS (A), salidroside (B), H_2_O_2_ (C), H_2_O_2_ + Salidroside (D). Scale bar: 200 μm.

**Supplementary Fig. S4**

**
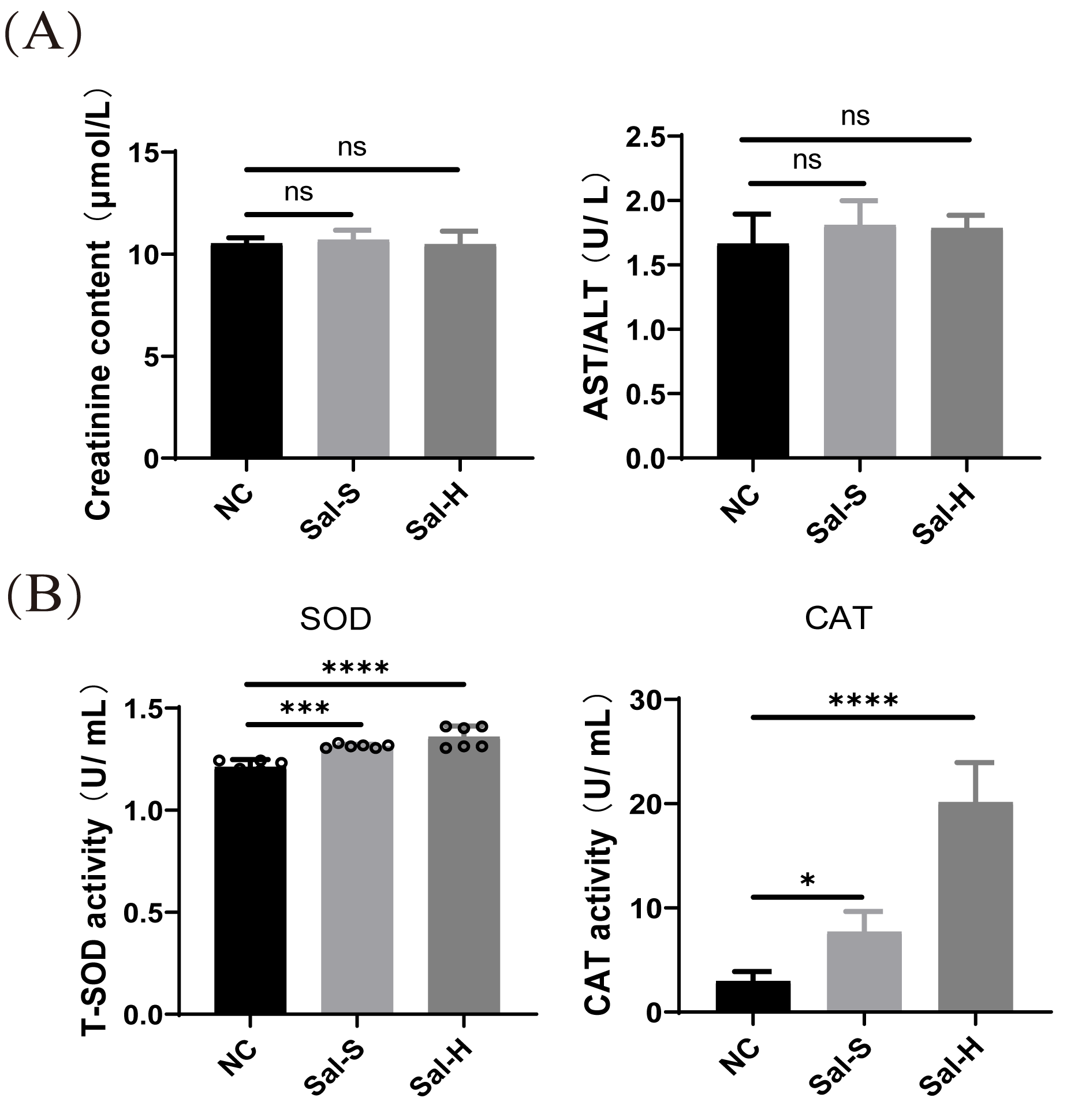
**

Supplementary Figure S4 The toxic effect and antioxidant of salidroside in mice. (A) After treatment with salidroside, the content of creatinine and the activity of AST/ALT were used to analyze the toxic effect of salidroside on mice serum in each group. (B) The levels of SOD and CAT in the mice serum were measured by the corresponding kits. All data are expressed as mean ± SD (n = 6). ns, no significance, **p* < 0.05, ****p* < 0.005, *****p* < 0.001.

AST: Aspartate aminotransferase, ALT: Alanine transaminase, SOD: Superoxide dismutase; CAT: CAT: catalase.

**Supplementary Fig. S5**


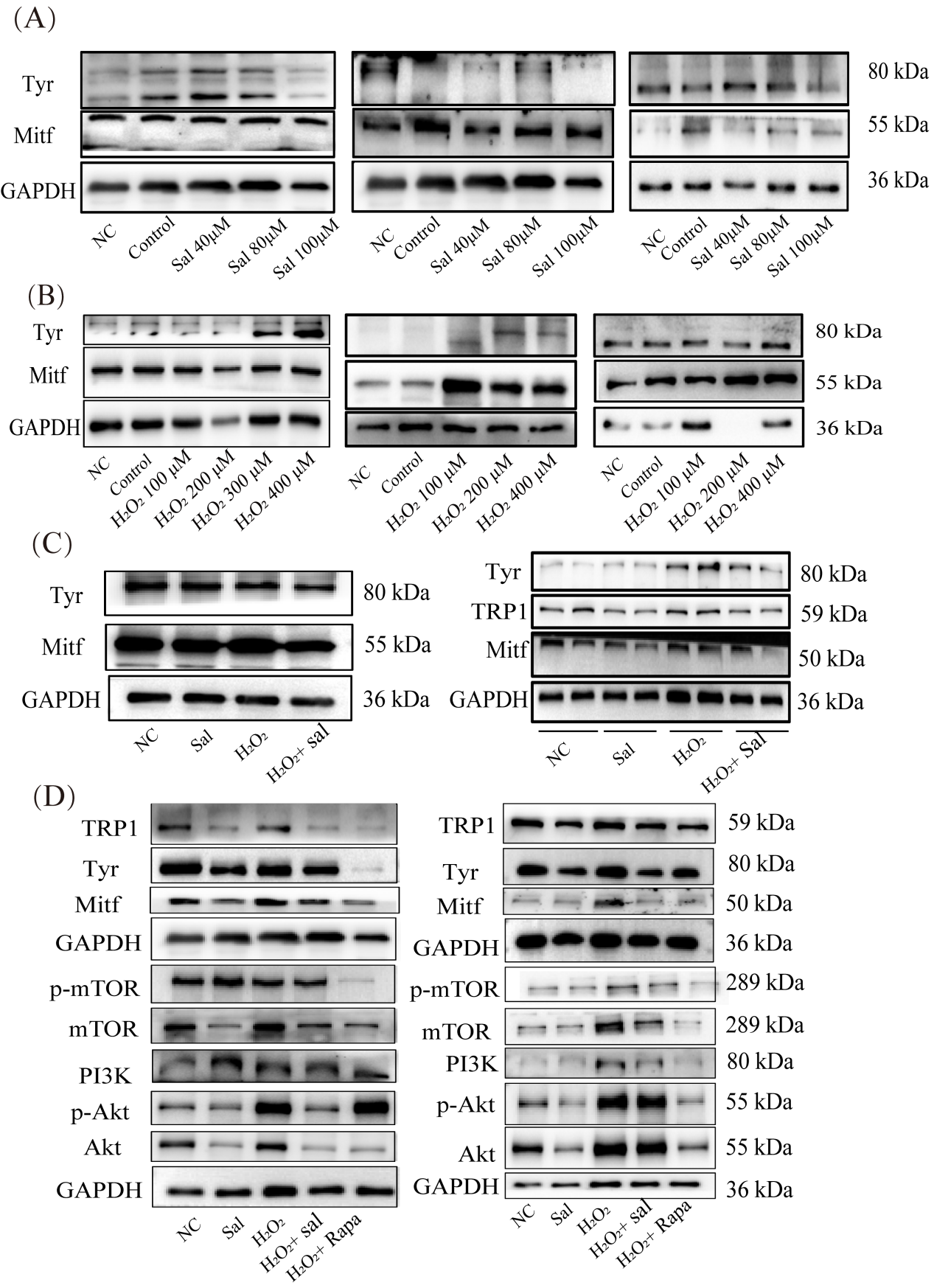


Supplementary Figure S5 Salidroside inhibited H_2_O_2_-inductd melanogenesis by inactivating PI3K/AKT/mTOR. (A) The protein-expression levels of Tyr and Mitf were examined by western blotting analysis and greyscale analysis of proteins. GAPDH functioned as a loading control, repeated three times. (B)The protein-expression levels of Tyr and Mitf were examined by western blotting analysis and greyscale analysis of proteins. GAPDH functioned as a loading control, repeated three times. (C) The protein expression of Tyr, TRP-1 and Mitf was examined by western blotting analysis, repeated one time. (D) The proteins expression levels of melanin synthesis-related proteins (TRP1, Tyr and Mitf) and PI3K/Akt/mTOR signaling pathway-related proteins (PI3K, Akt and mTOR) in B16F10, repeated two times.

TRP1: Tyrosinase related protein 1; mTOR: Mammalian target of rapamycin. p-mTOR: Phosphorylation-Mammalian target of rapamycin; PI3K: Phosphoinositide 3-Kinase; p-PI3K: Phosphorylation-phosphoinositide 3-Kinase; Akt: AKT Serine/Threonine Kinase 1/2/3; p-Akt: Phosphorylation-Akt.

**Supplementary Fig. S6**


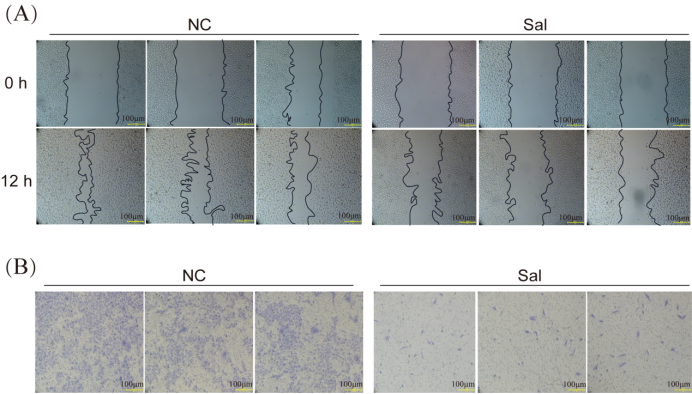


Supplementary Figure S6 Salidroside suppresses tumor growth in syngeneic tumor model. (A) Wounding healing assays in B16F10. Cells were treated with salidroside. (B) After 24 h of salidroside treatment, B16F10 cells was detected by Trans-well assay, scar = 100 μm.

**Supplementary Fig. S7**


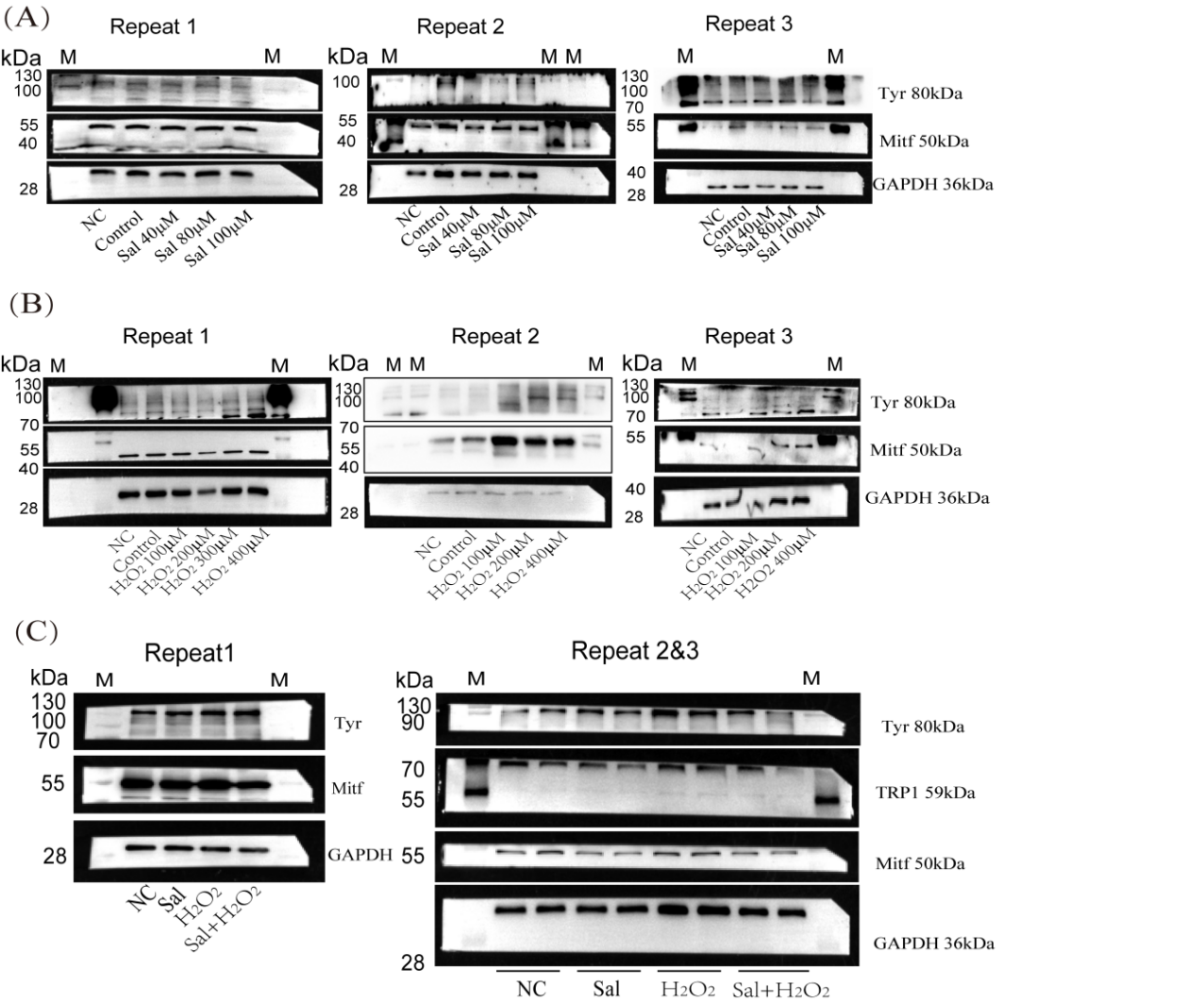

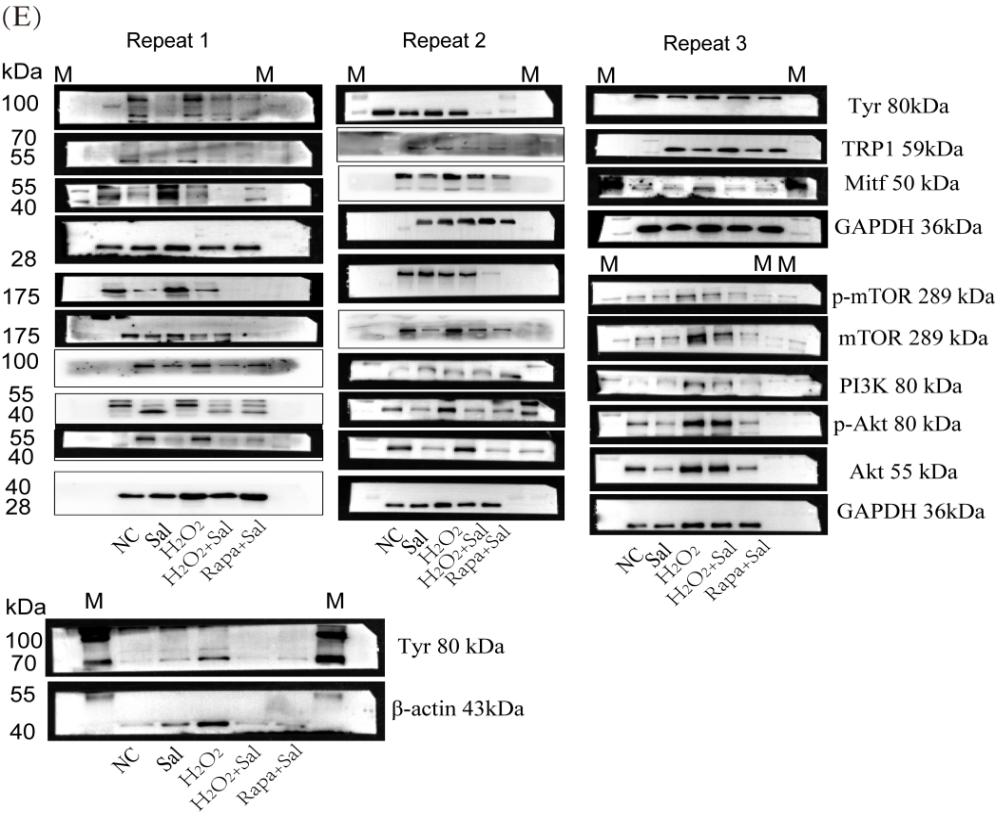


Supplementary Figure S7 The raw data of the western blotting images in the manuscript.
